# Within-breed and multi-breed GWAS on imputed whole-genome sequence variants reveal candidate mutations affecting milk protein composition in dairy cattle

**DOI:** 10.1186/s12711-017-0344-z

**Published:** 2017-09-18

**Authors:** Marie-Pierre Sanchez, Armelle Govignon-Gion, Pascal Croiseau, Sébastien Fritz, Chris Hozé, Guy Miranda, Patrice Martin, Anne Barbat-Leterrier, Rabia Letaïef, Dominique Rocha, Mickaël Brochard, Mekki Boussaha, Didier Boichard

**Affiliations:** 1 GABI, INRA, AgroParisTech, Université Paris Saclay, 78350 Jouy-en-Josas, France; 2 Institut de l’Elevage, 75012 Paris, France; 3 Allice, 75012 Paris, France

## Abstract

**Background:**

Genome-wide association studies (GWAS) were performed at the sequence level to identify candidate mutations that affect the expression of six major milk proteins in Montbéliarde (MON), Normande (NOR), and Holstein (HOL) dairy cattle. Whey protein (α-lactalbumin and β-lactoglobulin) and casein (αs1, αs2, β, and κ) contents were estimated by mid-infrared (MIR) spectrometry, with medium to high accuracy (0.59 ≤ R^2^ ≤ 0.92), for 848,068 test-day milk samples from 156,660 cows in the first three lactations. Milk composition was evaluated as average test-day measurements adjusted for environmental effects. Next, we genotyped a subset of 8080 cows (2967 MON, 2737 NOR, and 2306 HOL) with the BovineSNP50 Beadchip. For each breed, genotypes were first imputed to high-density (HD) using HD single nucleotide polymorphisms (SNPs) genotypes of 522 MON, 546 NOR, and 776 HOL bulls. The resulting HD SNP genotypes were subsequently imputed to the sequence level using 27 million high-quality sequence variants selected from Run4 of the 1000 Bull Genomes consortium (1147 bulls). Within-breed, multi-breed, and conditional GWAS were performed.

**Results:**

Thirty-four distinct genomic regions were identified. Three regions on chromosomes 6, 11, and 20 had very significant effects on milk composition and were shared across the three breeds. Other significant effects, which partially overlapped across breeds, were found on almost all the autosomes. Multi-breed analyses provided a larger number of significant genomic regions with smaller confidence intervals than within-breed analyses. Combinations of within-breed, multi-breed, and conditional analyses led to the identification of putative causative variants in several candidate genes that presented significant protein–protein interactions enrichment, including those with previously described effects on milk composition (*SLC37A1*, *MGST1*, *ABCG2*, *CSN1S1*, *CSN2*, *CSN1S2*, *CSN3*, *PAEP*, *DGAT1*, *AGPAT6*) and those with effects reported for the first time here (*ALPL*, *ANKH*, *PICALM*).

**Conclusions:**

GWAS applied to fine-scale phenotypes, multiple breeds, and whole-genome sequences seems to be effective to identify candidate gene variants. However, although we identified functional links between some candidate genes and milk phenotypes, the causality between candidate variants and milk protein composition remains to be demonstrated. Nevertheless, the identification of potential causative mutations that underlie milk protein composition may have immediate applications for improvements in cheese-making.

**Electronic supplementary material:**

The online version of this article (doi:10.1186/s12711-017-0344-z) contains supplementary material, which is available to authorized users.

## Background

In cattle, milk protein composition is mostly influenced by genetic factors [1–4] and is of interest because it determines cheese-making properties [5]. Bovine milk protein composition can be predicted at a large scale by analyzing mid-infrared (MIR) spectra, which is routinely performed [6, 7]. Combined with cow genotyping, this technique may open avenues to investigate the genomic regions that influence milk protein composition. In a previous genome-wide association study (GWAS) based on the bovine 50 K single nucleotide polymorphism (SNP) array, we highlighted numerous genomic regions with very significant effects on milk protein composition in the three main breeds of French dairy cattle: Holstein (HOL), Montbéliarde (MON), and Normande (NOR) [8]. However, because the 50 K SNP array contains only a small fraction of the total number of genomic variants, we were not able to directly pinpoint candidate mutations.

In Run4 of the 1000 bull genome reference population, a database containing more than 56 million SNPs and small insertions/deletions (InDel) was constructed by analyzing whole-genome sequences (WGS) from 1147 bulls representing 27 different breeds, including 288 HOL, 28 MON and 24 NOR bulls. These data can then be used to impute WGS from experimentally or routinely obtained 50 K SNP genotypes [9]. In this way, imputed WGS can be obtained for a large number of animals and in particular, those with phenotypes.

Since WGS contain almost all the genomic variants, they should contain the causal mutations for a given trait and, thus they provide a much higher GWAS resolution. However, due to the long-range linkage disequilibrium that exists within dairy cattle breeds, the resolution of within-breed GWAS is often limited. For causal mutations that are shared among breeds, a multi-breed model can be used to refine regions that harbour quantitative trait loci (QTL). This approach takes advantage of the historical recombination events that have occurred in each breed, resulting in linkage disequilibrium over shorter distances and better resolution [10].

Here, we report the results of a GWAS at the sequence level for six major milk protein contents, namely α-lactalbumin and β-lactoglobulin and αs1, αs2, β, and κ caseins from HOL, MON, and NOR cows. The results of within-breed, multi-breed, and conditional analyses, that fit the most significant variant in addition to other tested variants, are examined together in order to pinpoint potential candidate variants in each genomic region.

## Methods

### Animals, phenotypes, and genotypes

For this study, we did not perform any animal experiment, thus no ethical approval was required. Details on the animals and milk analyses are in Sanchez et al. [8]. Briefly, MIR spectra were obtained for 848,068 milk samples from 156,660 cows of the three main French dairy breeds: Montbéliarde (MON), Normande (NOR), and Holstein (HOL). These spectra were used to predict milk protein content (PC) and milk protein composition with the equations derived as described by Ferrand et al. [7]. More details about the method and the calibration population used are in Sanchez et al. [4]. The contents of the six main milk proteins (α_s1_-CN, α_s2_-CN, β-CN, κ-CN, α-LA, and β-LG) were predicted in g/100 g protein. Total casein content and total whey protein content were also analyzed ($\varSigma$-CN and $\varSigma$-WP, respectively). In order to adjust phenotypes for non-genetic effects, a within-breed mixed model was applied to test-day data using the GENEKIT software [11]. This single-trait repeatability model included genetic, permanent environmental, and residual random effects, as well as herd × test-day, parity × stage of lactation, year × month of calving, and spectrometer × test month fixed effects. We applied this model to data from the first two lactations that included at least three test-day records across lactations during the study period. Then, test-day data were corrected for all non-genetic effects included in the model and averaged per cow. Thus, for each trait and each cow, a single phenotype was defined and subsequently used in GWAS analyses. In total, 293,780, 58,594, and 72,973 test-day records were analyzed, which corresponded to 44,959 MON, 12,428 NOR, and 14,530 HOL cows, i.e. an average of 6.5, 4.7, and 5.0 test-day records per cow, respectively.

Among these cows, 8010 were genotyped with the Illumina BovineSNP50 BeadChip (Illumina Inc., San Diego). We applied the following quality control filters: the individual call rate had to be higher than 95%, the SNP call rate higher than 90%, the minor allele frequency (MAF) higher than 5%, and genotype frequencies had to be in Hardy–Weinberg equilibrium with *P* > 10^−4^. The final dataset included between 37,332 and 41,028 SNPs (Table 1), depending on the within-breed or multi-breed population considered, for 7907 cows (3032 MON, 2659 NOR, and 2216 HOL) with phenotypes.

**Table 1:** Features of the Montbéliarde (MON), Normande (NOR), Holstein (HOL), and multi-breed populations

Number of	MON	NOR	HOL	Multi-breed
Phenotyped cows	44,959	12,428	14,530	71,917
Total test-day records	293,780	58,594	72,973	425,347
Test-day records per cow	6.5	4.7	5	5.9
Genotyped cows	3032	2659	2216	7907
Polymorphic 50 K SNPs	37,332	37,690	39,158	41,028
Polymorphic HD SNPs	548,185	549,359	553,712	586,749
Polymorphic sequence variants	15,957,336	14,809,860	15,116,501	18,366,748
Sequence variants (MAF ≥ 2%)	11,755,172	11,445,432	11,592,432	13,534,013

### Imputation to whole-genome sequences

The 50 K SNP genotypes of the 7907 cows were imputed to whole-genome sequence (WGS) using FImpute software, which accurately and quickly analyzes large datasets [12]. A two-step approach was applied in order to improve the accuracy of results: from 50 to 777 K high-density (HD) SNPs, and then, from imputed HD SNPs to WGS [13]. All imputations were performed separately for each breed using either a breed-specific (from 50 K to HD SNPs) or a multi-breed (from HD SNPs to WGS) reference panel depending on the targeted density [14]. In each MON, NOR and HOL breed, imputations to the HD SNP level were performed using a within-breed reference population that included respectively 522 MON, 546 NOR, and 776 HOL bulls that had been genotyped with the Illumina BovineHD BeadChip (Illumina Inc., San Diego, CA). Around 550,000 SNPs were retained in each breed after removing SNPs that failed in the quality control filters, as described above for the 50 K (Table 1). WGS variants were imputed from HD SNP genotypes using WGS variants of the 1147 bulls from Run4 of the 1000 Bull Genomes consortium; these bulls represent 27 cattle breeds (see Additional file 1: Table S1), with 288 HOL, 28 MON, and 24 NOR individuals [9]. The protocol used was defined in the “1000 bull genomes” consortium [9]. Whole-genomes of all individuals were used for 2 × 100 bp paired-end sequencing using Illumina sequencing-by-synthesis technology and sequence reads were further filtered for quality and subsequently aligned to the UMD3.1 reference sequence, as previously described [9, 15]. Small genomic variations (SNPs and InDel) were detected using SAMtools 0.0.18 [16]. Raw variants were further filtered to produce 27,754,235 autosomal variants [15]. Filtered variants were subsequently annotated with the Ensembl variant effect predictor (VEP) pipeline v81 [17] and effect of the amino acid changes was predicted using the SIFT tool [18].

Precision of imputation from HD to sequence was assessed by comparing imputed genotypes with those obtained by re-genotyping a subset of the same cows with a custom chip. This additional information was not used in the imputation process. Two datasets were available: (1) a group of 168 Holstein cows that were genotyped with the first version (V1) of the EuroG10k Illumina chip, with 721 additional markers; and (2) a group of 2142 Montbéliarde cows that were genotyped with the fourth version (V4) of the same EuroG10k chip containing 3082 additional SNPs. Only SNPs with good technical quality (call rate > 95%, validation of the clusters by visual inspection, within-breed allelic frequency not significantly different across chip versions) were used. Imputation accuracy was measured by the squared correlation between true and imputed genotypes and by the genotypic and allelic concordance rate.

In order to remove SNPs with the lowest accuracies of imputation, only variants with a MAF higher than 0.02 were retained for further association analyses. Thus, about 11 million variants were included in each within-breed analysis and around 13 million were included in multi-breed analyses (Table 1).

### Whole-genome sequence association analyses

We performed single-trait association analyses between all the polymorphic variants and the nine measured milk protein composition traits: PC, α-LA, β-LG, α_s1_-CN, α_s2_-CN, β-CN, κ-CN, $\varSigma$-CN, and $\varSigma$-WP (Table 2).

**Table 2:** MIR predictions for milk protein composition in Montbéliarde (MON), Normande (NOR), and Holstein (HOL) cows

Trait		Accuracy^a^		Means ± standard deviations^b^		
		R^2^	RE	MON	NOR	HOL
PC	Protein content	1.00	0.73	3.4 ± 0.4	3.6 ± 0.4	3.3 ± 0.4
α-LA	α-lactalbumin	0.59	14.4	4.07 ± 0.28	4.16 ± 0.36	4.27 ± 0.42
β-LG	β-lactoglobulin	0.74	11.7	8.25 ± 1.12	7.94 ± 1.03	8.46 ± 1.17
α_s1_-CN	α_s1_-casein	0.88	4.7	27.8 ± 0.55	27.8 ± 0.68	27.9 ± 0.69
α_s2_-CN	α_s2_-casein	0.82	7.5	9.53 ± 0.30	9.89 ± 0.33	9.69 ± 0.39
β-CN	β-casein	0.92	3.7	36.6 ± 0.88	36.2 ± 1.2	36.2 ± 1.2
κ-CN	κ-casein	0.80	8.4	9.75 ± 0.60	9.87 ± 0.48	9.43 ± 0.58
$\varSigma$-CN	Sum of caseins	0.97	2.7	83.7 ± 0.94	83.7 ± 1.5	83.1 ± 1.4
$\varSigma$-WP	Sum of whey proteins	0.73	8.9	12.6 ± 1.1	11.9 ± 1.2	12.6 ± 1.3

^a^Accuracy of MIR predictions (R^2^ = coefficient of determination and RE = relative error) estimated by Ferrand et al. [7] for protein composition expressed as g/100 g milk

^b^g/100 g milk for protein content (PC) and g/100 g protein for other traits

All association analyses were performed using the *mlma* option of the GCTA software, which applies a mixed linear model that includes the candidate variant [19]:

1 $${\mathbf{y}} = {\mathbf{1}}\upmu + {\mathbf{x}}{\text{b}} + {\mathbf{u}} + {\mathbf{e}} ,$$

where **y** is the vector of pre-adjusted phenotypes, averaged per cow; $\upmu$ is the overall mean; b is the additive fixed effect of the candidate variant to be tested for association; **x** is the vector of imputed genotypes coded as 0, 1, or 2 (number of copies of the second allele); ${\mathbf{u}} \sim {\text{N}}({\mathbf{0}}, {\mathbf{G}}\upsigma_{\text{u}}^{2} )$ is the vector of random polygenic effects, with ${\mathbf{G}}$ the genomic relationship matrix (GRM), calculated by using the HD SNP genotypes [20], and $\upsigma_{\text{u}}^{2}$ the polygenic variance, estimated based on the null model $({\mathbf{y}} = {\mathbf{1}}\upmu + {\mathbf{u}} + {\mathbf{e}})$ and then fixed while testing for the association between each variant and the trait; and ${\mathbf{e}} \sim {\text{N}}({\mathbf{0}}, {\mathbf{I}}\upsigma_{\text{e}}^{2} )$ is the vector of random residual effects, with **I** the identity matrix and $\upsigma_{\text{e}}^{2}$ the residual variance. Within-breed, the number of test-day records did not differ very much across cows, thus, the residual variance was assumed to be constant across cows.

For multi-breed association analyses, Model (2) was applied by adding a fixed breed effect **v** to Model (1), with **W** as the incidence matrix relating phenotypes to breed effect (three levels), and **x**, **b**, **u**, and **e** as defined previously:

2 $${\mathbf{y}} = {\mathbf{Wv}} + {\mathbf{x}}{\text{b}} + {\mathbf{u}} + {\mathbf{e}} .$$

The Bonferroni correction was applied to the thresholds in order to account for multiple testing. A very stringent correction was used, which considered all 13 million tests as independent. Therefore, the 5% genome-wide threshold of significance corresponded to a nominal *P* value of 3.7 × 10^−9^ (−log_10_(*P*) = 8.4). QTL regions were identified by grouping significant results that were located within the same 2 million base-pair (Mbp) interval in a single genomic region, regardless of the breeds or traits under study. QTL regions were determined by considering positions of variants included in the upper third of the peak. For a given trait in a given breed, when two consecutive QTL regions had overlapping confidence intervals, or when the distance between the limits of the confidence intervals was less than 1 Mbp, only the confidence interval that presented the most significant results was retained.

### Conditional association analyses

In the most significant QTL regions, conditional analyses were carried out using the *cojo* option of GCTA [21] in order to conclude if multiple significant variants in a genomic region were due to LD with the same causal mutation or to the presence of multiple causal mutations. Association analyses were performed by including in the model the most significant variant or the putative causal mutation as a fixed effect and by testing all variants that were not in strong LD with the conditional variant (r^2^ < 0.9).

### Annotation and protein interactions

Sequence-derived polymorphisms were extracted for candidate mutation regions from the corresponding VCF files [22]. All variants with a −log_10_(*P*) higher than 8.4 and located within confidence intervals were annotated. To avoid missing important genes, confidence intervals were extended by 100 kb on each side.

In addition, functional protein–protein interactions (PPI) encoded by candidate genes were investigated, as well as gene ontology (GO) enrichment, using the STRING Genomics 10.0 database of protein–protein interaction (PPI) networks [23]. This database provides (1) known PPI from curated databases or experiments and (2) PPI predicted on the basis of gene neighborhood, gene fusions, gene co-occurrence, text mining in literature, co-expression, or protein homology. A global PPI network was constructed which retained only interactions with a high level of confidence (score > 0.4).

## Results

**Fig. 1:**
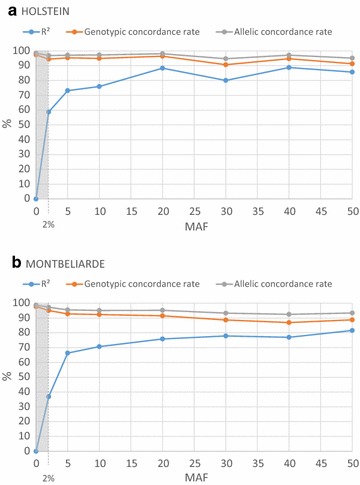
Precision of imputations at the whole-genome sequence level in **a** Holstein and **b** Montbéliarde breeds, according to MAF. The 2% limit corresponds to the MAF threshold for markers used in GWAS

The results of imputation accuracy at the sequence level for SNPs used in the GWAS analyses (MAF ≥ 2%) are in Table 3. Squared correlations between imputed and true genotypes in the validation set reached 76 and 84%, in Montbeliarde and Holstein breeds, respectively. This table also presents the overall results of concordance rate. Figure 1 shows the imputation precision according to MAF in the two breeds.

**Table 3:** Accuracies of imputations on whole-genome sequences in Holstein (HOL) and Montbéliarde (MON) breeds

Breed	HOL	MON
Number of cows	168	2142
EuroG10k chip version	V1	V4
Number of markers in the custom part	721	3082
Number of markers after quality control and MAF ≥ 0.02	221	1108
R^2^ (%)	83.7	76.1
Genotypic concordance rate (%)	93.7	89.7
Allelic concordance rate (%)	96.5	94.0

Among the 13 million tested variants, 71,755 had genome-wide significant effects (−log_10_(*P*) ≥ 8.4) in at least one within-or multi-breed analysis and for at least one milk protein composition trait.

**Fig. 2:**
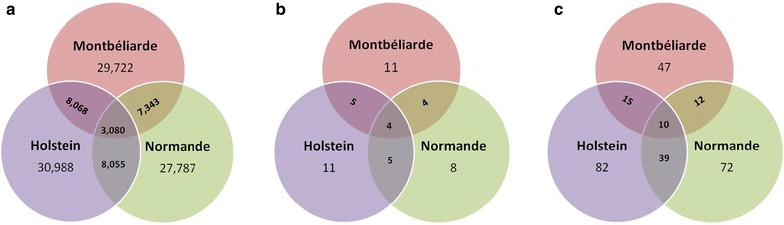
Number of overlapping **a** variants with genome-wide significant effects (−log_10_(*P*) ≥ 8.4), **b** QTL regions, and **c** genes containing variants with genome-wide significant effects among Montbéliarde, Normande, and Holstein breeds

Among these, 29,722, 27,787, and 30,988 were found in within-breed MON, NOR, and HOL analyses, respectively. Some of these variants had significant effects in multiple breeds: 7343 in both MON and NOR, 8055 in NOR and HOL, 8068 in HOL and MON, and 3080 in all three breeds (Table 4; Fig. 2a).

**Table 4:** Number of variants with genome-wide significant effects (−log_10_ *(P)* > 8.4) for milk composition traits in within-and multi-breed analyses

Trait	Within-breed analyses				Multi-breed analyses
	MON^a^	NOR^a^	HOL^a^	Shared among three breeds	
PC	1905	1201	2394	0	2350
α-LA	4590	6490	8248	213	7224
β-LG	19,952	16,048	15,517	2266	18,612
α_s1_-CN	2232	708	629	182	2280
α_s2_-CN	866	193	636	1	1947
β-CN	665	734	524	96	1652
κ-CN	4110	5878	6532	553	7012
$\varSigma$-CN	13,920	8716	11,833	961	12,698
$\varSigma$-WP	16,583	13,126	15,327	1916	16,546
Total number of distinct variants	29,722	27,787	30,988	3080	34,248

^a^Montbéliarde (MON), Normande (NOR), and Holstein (HOL) cows

For each trait, the number of significantly associated variants was relatively consistent between breeds. It was lower (from 193 to 2394) for α_s2_-CN, β-CN, α_s1_-CN, and PC; higher (from 8716 to 19,952) for β-LG, κ-WP, and $\varSigma$-CN; and intermediate (from 4110 to 8248) for α-LA and κ-CN. Among these variants, 0 (PC) to 2266 (β-LG) were shared among the three breeds. Multi-breed analyses were more powerful, and detected a larger number of distinct variants with significant effects (34,248) than any of the within-breed analyses. However, the number of variants detected per trait was larger in one of the within-breed analyses than in the multi-breed analysis for PC, α-LA, β-LG, $\varSigma$-CN, and $\varSigma$-WP (Table 4), probably because of the long-range within-breed LD.

QTL regions were defined by merging the overlapping QTL regions obtained for the different traits and breeds and by grouping the corresponding significant results. Confidence intervals of these regions were defined as described in the Methods section. Thus, 34 QTL regions with significant effects on one or several milk protein composition traits were identified in within-breed and/or multi-breed analyses (see Additional file 2: Table S2). Three of these, located on chromosomes 6, 11, and 14, had significant pleiotropic effects on almost all protein composition traits analyzed (see Additional file 3: Table S3), while most (31 QTL) generally affected only one trait (see Additional file 4: Table S4).

The 34 QTL were distributed on 17 of the 29 bovine autosomes, with one to seven QTL per chromosome. Almost all of them (31) were detected in multi-breed analyses while 11, 8, and 11 QTL regions were found in MON, NOR, and HOL within-breed analyses, respectively. Four QTL regions, located on *Bos taurus* chromosome BTA6 (two regions at 45.8–46.9 Mbp and 85.2–87.4 Mbp), BTA11 (103.3 Mbp), and BTA20 (58.3–58.4 Mbp), were detected in three breeds. One additional region on BTA29 at about 9.6 Mbp was common to MON and HOL, and another region on BTA14 at 1.7–1.8 Mbp was common to HOL and NOR (Fig. 2b). The six QTL shared between two or three breeds had the most significant effects, along with one QTL detected only in the NOR breed on BTA2, at 131.8 Mbp (−log_10_(*P*) ≥ 20; *P* value < 10^−13^ after Bonferroni correction).

Multi-breed analyses led to the detection of a larger number of QTL regions than within-breed analyses: 14 of the 31 QTL detected in multi-breed analyses were not found in within-breed analyses. For the 17 QTL regions found in both within-and multi-breed analyses, the −log_10_(*P*) value of the most significant (top) variant was almost always higher in multi-than in within-breed analyses; this was true even for most of the regions that had significant effects in only one within-breed analysis. For these QTL, the mean −log_10_(*P*) value of the most significant (top) variant was 64 in multi-breed analyses versus 49, 46, and 42 in MON, NOR and HOL within-breed analyses, respectively. In addition, the QTL confidence intervals generated by multi-breed analyses contained a smaller number of variants than those produced by within-breed analyses. For the 17 QTL regions, an average of 134 variants (2–374) were found in multi-breed analyses versus 189 (39–335), 287 (61–872), and 308 (9–1236) in MON, NOR, and HOL within-breed analyses, respectively. However, in some QTL regions, specifically those located on BTA2 (131.8 Mbp), 6 (38 Mbp), and 19 (61 Mbp), the number of significant variants was smaller in within-breed analyses than in the multi-breed analysis.

**Fig. 3:**
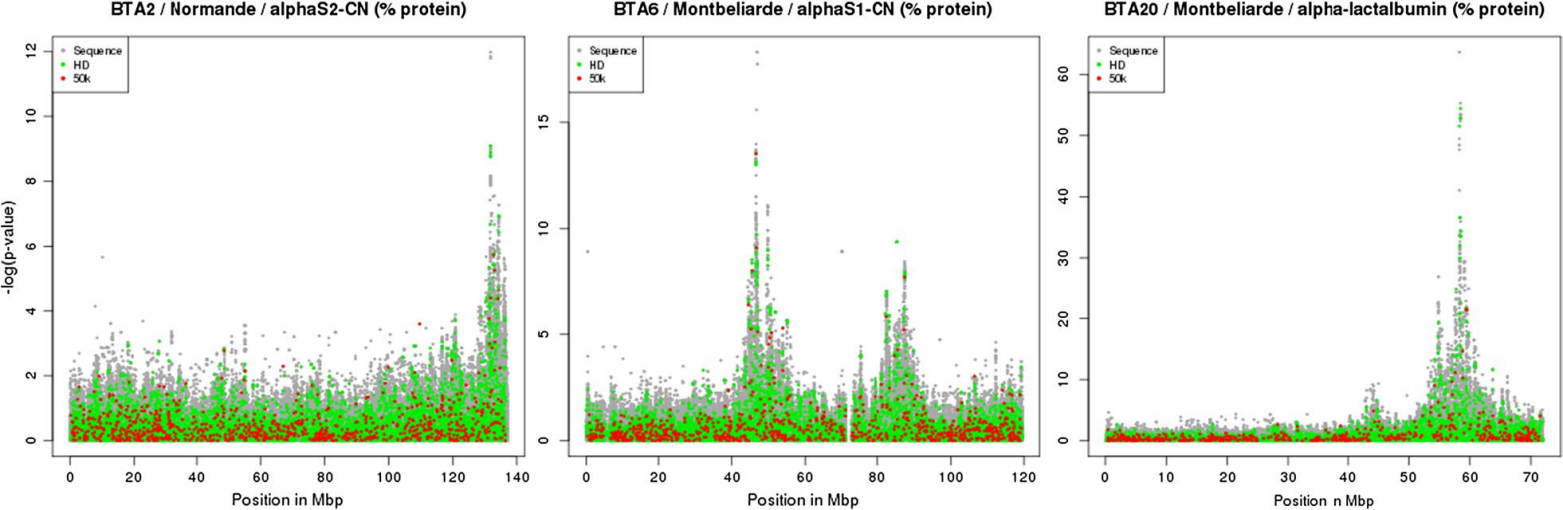
−log_10_(*P*) plotted against the position of variants on BTA2, 6, and 20 using three SNP densities: 50 K, HD, and whole-genome sequence SNPs

Manhattan plots of three of the most significant QTL regions are in Fig. 3 for the three densities of markers (50 K SNP, HD SNP, or sequence). In each of these regions, several peaks are detected with the WGS data, whereas with the 50 K SNP density and in some cases with the HD SNP density, only one peak was observed.

All variants included within confidence intervals (+100 kb on each side) were functionally annotated (Table 5) and (see Additional file 5: Table S5). The percentage of variants that were located within genes ranged from 60.5% in HOL to 73.4% in NOR within-breed analyses, and it was intermediate in multi-breed analyses (65.8%). The vast majority of the genic variants were located within introns and in upstream or downstream regions. A total of 25, 82, 72, and 56 missense variants were found in MON, NOR, HOL, and multi-breed analyses, respectively; among these, we detected the previously reported missense mutations in the *PAEP* (103,303,475 bp) and *DGAT1* (1,802,266 bp) genes.

**Table 5:** Functional annotations of variants included within confidence intervals (±100 kb) of the 34 QTL in the three within-and multi-breed analyses

Functional annotation	Within-breed analyses			Multi-breed analyses
	MON^a^	NOR^a^	HOL^a^	
Intergenic	1514	1465	2676	1971
Intronic	1079	1804	1937	1737
3′ UTR	11	14	69	35
5′ UTR	14	27	16	18
Downstream	710	988	1276	1159
Inframe insertion	0	0	1	0
Missense	25	82	72	56
Splice acceptor	0	0	3	0
Synonymous	30	114	118	91
Upstream	509	1009	612	685
% genic	61.1	73.4	60.5	65.8
% genic non intronic	33.4	40.6	32.0	35.5

^a^Montbéliarde (MON), Normande (NOR), and Holstein (HOL) cows

In 29 QTL regions, annotation led to the identification of candidate genes for milk protein composition. In total, 47, 72, and 82 candidate genes were identified in MON, NOR, and HOL within-breed analyses (109 in multi-breed analyses). Some of these were shared across breeds: 12 were found in both MON and NOR, 15 in MON and HOL, 39 in HOL and NOR, among which 10 were common to the three breeds (Fig. 2c). However, within a given region, the top variant was always different among the different breeds. The top variant was located in a gene in 21 of these regions, while in the remaining eight regions, the top variant was intergenic. However, these eight regions contained other variants located within confidence intervals that were annotated in genes, and of these, the most significant one was denoted the top genic variant. Genic variants with the most significant results were located within intron regions for 15 QTL and mainly upstream or downstream regulatory regions for 14 QTL. In total, 22 genes were identified as the best candidates to explain the majority of the variability of milk protein composition in MON, NOR, and HOL cows. They were located on BTA1 (*SLC37A1*), BTA2 (*ALPL*), BTA5 (*MGST1*), BTA6 (*ABCG2*, *MEPE*, *PKD2*, *HERC3*, *SEPSECS*, *SEL1L3*, *DHX15*, *CSN1S1*, *CSN2*, *CSN1S2*, and *CSN3*), BTA11 (*PAEP*), BTA14 (*DGAT1*, *RECQL4*, *MROH1*, and *BOP1*), BTA20 (*ANKH*), BTA27 (*AGPAT6*), and BTA29 (*PICALM*).

**Fig. 4:**
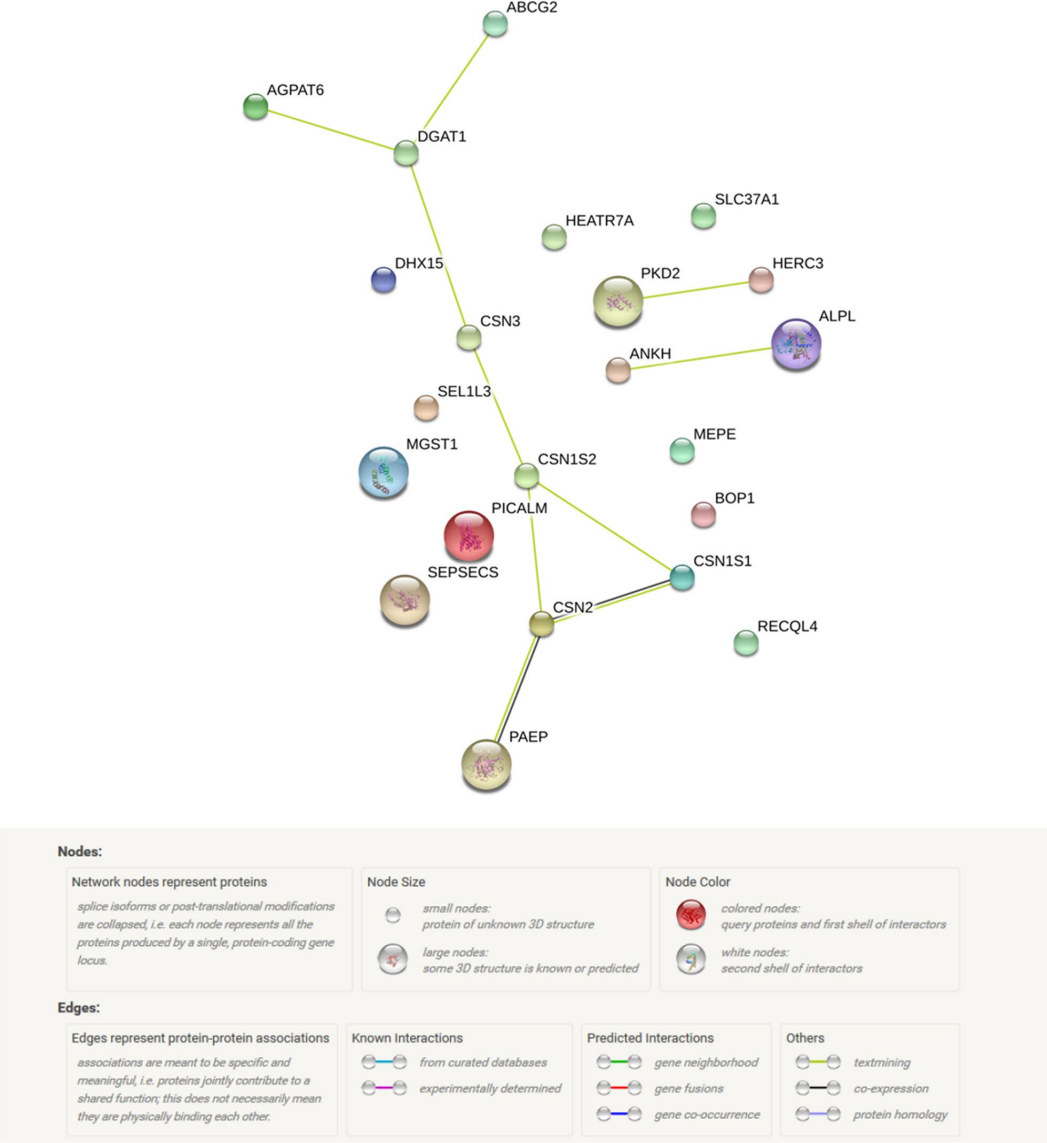
Protein network of the 22 most probable candidate genes detected, according to STRING v10.0 action view

Protein–protein interactions (PPI), as well as GO enrichment, were investigated for the 22 most plausible candidate genes of our study. Network proteins encoded by these genes had significantly more interactions than expected (10 edges identified; PPI enrichment *P* value = 3.4 × 10^−9^; Fig. 4), while GO terms for 12 biological processes, seven cellular components, and one molecular function were significantly (FDR < 0.05) enriched with two to nine of these genes for milk protein composition (Table 6).

**Table 6:** Gene Ontology (GO) functional enrichment with false discovery rate (FDR) < 0.05

	Pathway ID	Pathway description	Gene count	FDR	Genes
Biological process	GO.1903494	Response to dehydroepiandrosterone	4	1.73e–08	*CSN1S1, CSN1S2, CSN2, CSN3*
	GO.1903496	Response to 11-deoxycorticosterone	4	1.73e–08	*CSN1S1, CSN1S2, CSN2, CSN3*
	GO.0032570	Response to progesterone	4	1.81e–07	*CSN1S1, CSN1S2, CSN2, CSN3*
	GO.0097305	Response to alcohol	5	3.69e–07	*ALPL, CSN1S1, CSN1S2, CSN2, CSN3*
	GO.0032355	Response to estradiol	4	2.34e–06	*CSN1S1, CSN1S2, CSN2, CSN3*
	GO.1901700	Response to oxygen-containing compound	6	9.04e–05	*ALPL, CSN1S1, CSN1S2, CSN2, CSN3, PKD2*
	GO.0014070	Response to organic cyclic compound	5	0.000176	*ALPL, CSN1S1, CSN1S2, CSN2, CSN3*
	GO.0033993	Response to lipid	5	0.000181	*ALPL, CSN1S1, CSN1S2, CSN2, CSN3*
	GO.0009719	Response to endogenous stimulus	5	0.00205	*CSN1S1, CSN1S2, CSN2, CSN3, PKD2*
	GO.0048732	Gland development	3	0.0281	*CSN2, CSN3, PKD2*
	GO.0060416	Response to growth hormone	2	0.0281	*CSN1S1, CSN1S2*
	GO.0007595	Lactation	2	0.0298	*CSN2, CSN3*
Cellular component	GO.0005796	Golgi lumen	4	1.97e–08	*CSN1S1, CSN1S2, CSN2, CSN3*
	GO.0012505	Endomembrane system	8	0.00253	*AGPAT6, CSN1S1, CSN1S2, CSN2, CSN3, DGAT1, MGST1, PKD2*
	GO.0005576	Extracellular region	7	0.0372	*ALPL, CSN1S1, CSN1S2, CSN2, CSN3, PAEP, PKD2*
	GO.0005789	Endoplasmic reticulum membrane	4	0.0372	*AGPAT6, DGAT1, MGST1, PKD2*
	GO.0042175	Nuclear outer membrane-endoplasmic reticulum membrane network	4	0.0372	*AGPAT6, DGAT1, MGST1, PKD2*
	GO.0044444	Cytoplasmic part	9	0.0372	*ABCG2, AGPAT6, CSN1S1, CSN1S2, CSN2, CSN3, DGAT1, MGST1, PKD2*
	GO.0044446	Intracellular organelle part	9	0.0372	*ABCG2, AGPAT6, CSN1S1, CSN1S2, CSN2, CSN3, DGAT1, MGST1, PKD2*
Molecular function	GO.0035375	Zymogen binding	2	0.0177	*CSN1S2, CSN3*

## Discussion

In this paper, we report the results of a whole-genome sequence scan for milk protein composition predicted from MIR spectra. We conducted within-and multi-breed analyses using imputed WGS of 7907 cows from three French dairy breeds. This approach led to the detection of 34 distinct regions that affect the protein composition of milk. The use of imputed WGS enabled us to confirm 22 of the 39 QTL that were previously detected from 50 K SNP genotypes [8] and to identify 12 novel QTL. In addition to genetic parameter results [4] and QTL detection results with the 50 K chip [8], these results confirm that MIR predictions are sufficiently accurate for genetic investigations. Repeated test-day records compensated for the moderate MIR prediction accuracy of some proteins.

Seventeen QTL that had been detected with 50 K SNP genotype data were not found with imputed WGS, possibly because different methods were used in the two studies (linkage disequilibrium and linkage analysis in the 50 K SNP study versus GWAS in the current imputed-WGS study) and also possibly because of the more stringent significance thresholds applied here. For GWAS on WGS data, the very stringent threshold that we used (with Bonferroni correction considering all variants as independent) probably reduced the detection power but minimized the number of false positive QTL.

Instead, the better resolution of the WGS data, combined with the power of the multi-breed GWAS approach, led to the detection of 12 QTL that were not previously found in the 50 K SNP study. To evaluate the impact of marker density on GWAS results, we extracted 50 K and HD GWAS results from the WGS results. In several genomic regions, for example the regions on BTA2, 6, and 20 (Fig. 3), the increased resolution of the WGS data clearly makes it possible to identify two or more peaks whereas analysis of the 50 K SNP data detected only one peak.

Furthermore, the WGS resolution enables the use of a multi-breed approach, which is expected to better estimate the effects of rare variants and to reduce LD between neighboring variants. With the multi-breed analysis, we detected 14 QTL that were not detected in any of our within-breed analyses (see, for example, regions of the *MGST1* and *AGPAT6* genes described below). For QTL that were detected in both within-and multi-breed analyses, the multi-breed approach provided smaller confidence intervals of the QTL than within-breed analyses. The three French breeds used in our study are not strongly related. Based on 50 K SNP data, Gautier et al. [24] reported a partitioning of the genetic diversity of cattle into distinct groups of breeds with high geographical consistency. The three breeds were classified into three distinct groups: from Eastern France and Alps for MON, from Northern European for HOL and from the Channel Islands and Northwestern France for NOR. Thus, our results illustrate the extent to which a multi-breed approach can complement and enhance the information gained from within-breed analyses even if breeds pooled in multi-breed analyses have different genetic origins.

In a previous study [25], the imputation from 50 K to HD SNP densities was found to be very accurate in all three breeds with the number of HD genotypes used here (>500) in calibration. For the second imputation step, from HD SNPs to WGS, we used the Run 4 reference population of the 1000 Bull Genomes consortium, which contained 1147 bulls, of which 288 were HOL, 28 were MON, and 24 were NOR. Due to the larger number of sequenced HOL bulls compared to the two other breeds, imputation is more accurate with HOL data than with MON data. In NOR, we anticipate that imputation accuracy is close to that obtained in MON due to similar population structures and similar numbers of whole-genome sequences for major ancestors in both breeds. These results are in agreement with or are better than those already published in cattle. Daetwyler et al. [9] showed that the use of the 1000 Bull Genome multi-breed population (Run 2, 234 bulls) led to a similar imputation accuracy among data obtained from Holstein–Friesian, Fleckvieh, and Jersey cattle (near 80% of correlation) in spite of differences in the number of bulls in the reference population (129 Holstein–Friesian, 43 Fleckvieh, and 15 Jersey). Among the *PhénoFinlait* cows genotyped with the 50 K SNP Beadchip and then imputed to WGS, 1077 MON, 238 NOR, and 498 HOL originated from nine MON, five NOR and eight HOL bulls with WGS available in the Run4 reference population, i.e. 36, 9 and 22% of the PhénoFinlait cows, respectively. As expected, imputation accuracy dropped for variants with a low MAF. In order to limit the impact of imputation errors on the GWAS results, variants with a MAF lower than 2% were discarded from the analyses and almost all the genetic variants proposed as candidate variants in this study have moderate to high MAF.

Combining within-breed, multi-breed, and conditional GWAS analyses with functional annotations appears to be a good strategy for the differentiation of shared and breed-specific QTL. This approach also enables the direct identification of candidate genes with a very small number of candidate variants, or even in some cases, one unique variant which appears to be the best candidate to explain the observed effects.

On average, depending on the breed, between 60 and 73% of the QTL variants that we detected in the GWAS were located in genes; this is about twice as high as the percentage of genic variants at the whole-genome scale (35%; [15]). The most significant variants were located in 49 distinct genes, of which 22 were of particular interest, either because they were found in more than one breed or associated with several traits, or because they were previously described as influencing milk composition. These 22 genes, which are located in 11 distinct genomic regions and present significant protein–protein enrichment, are the most plausible candidates to explain a large part of the variation in milk protein composition among MON, NOR, and HOL cows. In four genomic regions (on BTA1, 2, 11, and 27), we identified one unique candidate variant (or a few candidate variants in LD) shared by all three breeds (in the *SLC37A1*, *ALPL*, *PAEP*, and *AGPAT6* genes, respectively). In three other genes, we suggest the presence of a breed-specific candidate variant (*MGST1* on BTA5 and *PICALM* on BTA29) or several candidate causative variants (*ANKH* on BTA20). Finally, four regions, including the *DGAT1* region on BTA14 and three regions on BTA6 (*ABCG2* region, a region at about 46 Mbp, and the casein gene cluster), were more complex, because they contained several candidate genes, each with several candidate variants. Eight of these candidate genes (*SLC37A1*, *MGST1*, *CSN1S1*, *CSN2*, *CSN1S2*, *CSN3*, *PAEP*, and *ANKH*) are known to be overexpressed in the mammary gland compared to other 17 tissues [26] and between two and nine of them are associated with one of the 20 GO terms in our study. The next sections describe these regions in more detail.

### *SLC37A1* (BTA1) and αs1-CN/ α-LA

The *SLC37A1* (*solute carrier family 37, member A1*) gene, which encodes a glucose-6-phosphate transporter that is involved in the homeostasis of blood glucose, is highly expressed in the mammary gland [27]. It could be a good candidate gene to explain the effects of the QTL identified on BTA1 on αs1-CN in both MON and multi-breed analyses and on the α-LA phenotype in the multi-breed analysis. In total, 138 distinct variants of this gene were located within the confidence intervals of the QTL, of which 133 were intronic, two were synonymous, and three were downstream (see Additional file 6: Figure S1a). For the αs1-CN/MON, αs1-CN/multi, and α-LA/multi results, the 80, 81, and 74 most significant variants in the peaks, respectively, were in intronic regions of *SLC37A1*. One downstream variant was detected for αs1-CN in the MON analysis, which ranked 104th among the significant variants, while multi-breed analyses revealed three downstream variants that ranked 81st, 87th, and 103rd. All intronic variants that are located at the top of the peaks are in strong LD but only one variant (indel), located at 144,397,274 bp, was common to all three TOP10 lists; it was 1st in the αs1-CN/MON ranking, 9th in the αs1-CN/multi-breed ranking, and 4th in the α-LA/multi-breed ranking. The top1 intronic variant detected in the αs1-CN/multi-breeds analysis, at 144,398,814 bp, ranked 75th in the αs1-CN/MON peak and 76th in the α-LA/multi-breed peak.

Two previous studies described the effects of *SLC37A1* gene variants on milk production traits. In an analysis of HD SNP genotypes, Kemper et al. [27] described six variants that are located between 144.325 and 144.525 Mbp in this region; the variant with the most significant effect was located in an intronic region of the gene (144,414,936 bp). In our study, this variant was included within the confidence interval of the QTL detected by the multi-breed analysis (−log_10_(*P*) = 10.2), but it ranked 101st. Two other intronic variants in strong LD in the *SLC37A1* gene, at 144,367,474 and 144,377,960 bp, were previously proposed as the best candidate mutations for changes in phosphorus concentration and milk production traits [28]. However, in our study in spite of relatively high MAF values (from 0.30 to 0.41 depending on the breed), these variants had a −log_10_(*P*) value lower than 6 for all analyzed traits. In another study of targeted QTL regions after imputation to WGS level, the variant with the most significant effects was located at 144,381,564 bp [29]. This variant is close to the candidate variant identified in our analysis, but it can be excluded as the causal variant in our populations since it is monomorphic in the MON, NOR, and HOL individuals analyzed here.

The conditional analyses that we performed included the two best candidate variants as well as the candidate variant described by Kemper et al. [27]. These revealed that including the variant located at 144,398,814 bp in the model completely removed the original signal while with each of the two other variants, a less significant peak persisted (see Additional file 6: Figure S1a). This variant, which has contrasting effects on αs1-CN and α-LA phenotypes, but with a more marked effect on the former, therefore constitutes the most probable candidate variant for the effects detected in our study.

### *ALPL* (BTA2) and αs2-CN

The QTL identified on BTA2 at 131.8 Mbp had significant effects on several traits (αs2-CN, β-CN, and κ-CN). In particular, although the αs2-CN-associated peaks were detected in all within-and multi-breed analyses, even if in the MON and HOL analyses, the maximal −log_10_(*P*) values did not reach the stringent threshold of 8.4 that we applied in this study (7 and 6.9, respectively; see Additional file 6: Figure S1b). In all analyses, the most significant variants were located in intronic regions of the *ALPL* (*alkaline phosphatase*) gene, which encodes a member of the alkaline phosphatase family of proteins. The most significant variant differed among the three within-breed analyses: it was located at 131,806,882 bp in NOR, 131,850,456 bp in MON, and 131,808,301 bp in HOL sequences. Instead, the top-ranked variant in the peak detected in the multi-breed GWAS was located at 131,806,882 bp. All three single-breed conditional analyses that included each of these variants as fixed effects lacked peaks (see Additional file 6: Figure S1b). These results suggest that all three intronic variants are in strong LD in the three breeds and that the causal mutation could be shared among breeds. Among all the variants at the top of the peaks, the intronic variant at 131,806,882 bp appears to be the most probable candidate variant in the *ALPL* gene for the observed effects on αs2-CN content; it ranked 1st, 6th, 26th, and 1st in the NOR, MON, HOL, and multi-breed peaks, respectively.

### *MGST1* (BTA5) and milk protein content (PC)

One region on BTA5 that contains 63 variants affected PC in the multi-breed analysis. The MON and NOR within-breed analyses revealed no peaks (−log_10_(*P*) < 6), whereas the HOL analysis detected a single peak with a −log_10_(*P*) = 8, which was close to the significance threshold of 8.4. Only one gene, *MGST1* (*microsomal glutathione S*-*transferase 1*), was present within the confidence interval obtained in the multi-breed analysis. Fifty-one variants were located in intronic (29), exonic (1 synonymous), 5′-UTR (2), or regulatory (19 in the upstream region) regions of the gene. The variant with the most significant effects was located at 93,950,211 bp in the upstream region and its −log_10_(*P*) value was 9.3, versus a value of 8.0 for the variants that ranked 2nd (93,950,116 bp and 93,950,288 bp), which were located, respectively, in the 5′-UTR and upstream regions of the gene. The MAF value for these variants was low in the MON population (0.006; <MAF threshold of 0.02) and ranged from 0.08 to 0.12 in NOR, from 0.37 to 0.42 in HOL, and from 0.19 to 0.22 in the multi-breed population. Thus, the fact that peaks were detected only in HOL (close to significance) and multi-breed (significant) analyses could be due to the relatively low MAF for these variants in MON and NOR. The most significant variants in our study are located near a variant that was reported by Raven et al. [29] to be responsible for changes in fat percentage in Holstein cows (at 93,951,731 bp (upstream) and ranked 23rd in our study) and also near variants previously linked to fat yield by Iso-Touru et al. [30] and Van den Berg et al. [31] (93,945,694 and 93,945,738 bp, respectively; both were intronic variants and were not significant here). Conditional analyses including each of the six variants as a fixed effect showed that all variants except those reported by Iso-Touru et al. [30] and Van den Berg et al. [31] explained the effects observed in our study (see Additional file 6: Figure S1c). Thus, the effects observed on fat content by Raven et al. [29] and on protein content in our study could be explained by the same causative variant. Recently, Littlejohn et al. [32] confirmed that *MGST1* has causative pleiotropic effects on milk composition (percentage and yield of fat, protein, and lactose). These authors failed to identify causative variants in the gene but they pointed to a cluster of 17 variants that were grouped in a 10-kbp segment of the *MGST1* gene (93,944,937–93,954,751). Only one of these 17 variants is located in the confidence interval of the QTL that we detected and this is an intronic variant (93,949,810 bp) that ranked 7th in the peak in spite of having a higher MAF (0.32) than the most significant variants (MAF = 0.19–0.22). Thus, our study highlights three new candidate mutations in the *MGST1* gene, which are located very close to each other, in the 5′-UTR region (93,950,116 bp) or in the upstream region (93,950,211 and 93,950,288 bp) of the gene.

### *ABCG2*, *MEPE*, *PKD2*, and *HERC3* (BTA6) and αs1-CN

Several QTL were found on BTA6. The first one, detected in HOL and multi-breed analyses, was located in the 37.6–38.4 Mbp region, which contains the Y581S polymorphism of the *ABCG2* gene (38,027,010 bp) that was described by Cohen-Zinder et al. [33] as a causative mutation for changes in milk yield and composition. This missense variant had MAF values of 0.0029 and 0.0018 in HOL and multi-breed populations, respectively, and therefore did not pass the MAF filter in both analyses. In spite of a low MAF, the Y581S polymorphism had a highly significant effect on the αs1-CN phenotype in both HOL and multi-breed analyses, with −log_10_(*P*) values of 31 and 21, respectively; these values were higher than those of the top variant in the peaks after filtering for MAF (20 and 15, respectively). However, among the sires of the HOL cows, six bulls were previously found to be heterozygous for the QTL detected in this region, but homozygous for this mutation [8]. Thus, we suggest that other mutations could be responsible for the QTL that affects milk protein composition.

In the HOL analysis, nine variants with MAF ranging from 0.022 to 0.041 were located within the confidence interval of the QTL. The most significant variants were located in intronic regions of the *ABCG2* gene, at 38,015,146 and 38,020,110 bp. Other variants, which are located in three other genes, i.e. *MEPE* (one downstream), *PKD2* (one intronic), and *HERC3* (two intronic), also had highly significant effects on αs1-CN. Due to the relatively low MAF of the candidate variants located in this region, these results require further analyses, including a larger number of animals and more accurate imputation or direct genotyping.

### *SEL1L3*, *SEPSECS*, and *DHX15* (BTA6) and αs1-CN

In all within-breed and multi-breed analyses, the αs1-CN phenotype was affected by another region of BTA6 at 45.8–46.9 Mbp. However, the most likely candidate genes differed among breeds. In MON, the nine variants with the most significant effects were located in intronic regions of the *SEL1L3* gene (max. at 46,874,151 bp). In NOR, the top 116 variants in the peak were intergenic, while the genic variant with the most significant effects was located in an intron of the *SEPSECS* gene (46,277,697 bp). In HOL, the most significant genic variants (*DHX15*) ranked 16th in the peak (45,639,181 and 45,640,564 bp). Finally, among the top 80 variants detected by the multi-breed analysis, only one was genic, which was located in an intron of the *SEL1L3* gene (46,874,514 bp, ranked 3rd in the MON analysis). There is insufficient concordance among these results to propose a single set of candidate variants.

### Pleiotropic effects of the casein gene region (BTA6)

On BTA6, we found a QTL that affected both the overall protein content of milk and the content of all four individual caseins in all three breeds. Variants with the most significant effects were located in an 840-kb interval that contains the 250-kb casein gene cluster (87,062,878–87,903,002 bp); other variants with effects on αs1-CN and β-CN in MON were located at 85.2 Mbp. In all within-and multi-breed analyses, the most significant effects were detected for the κ-CN phenotype, followed by αs1, αs2, or β-CN depending on the breed. In each analysis, the variant with the most significant effects on κ-CN was located within or in the immediate vicinity of the *CSN3* gene, which encodes the κ casein: at 87,376,747 bp (upstream) in NOR, 87,392,592 bp (5′-UTR) in MON and multi-breed, and 87,394,293 bp (downstream) in HOL. Each of these variants, as well as the κ casein A/B variant (87,390,576 bp, missense), was therefore included as a fixed effect in the conditional analyses. The results were breed-specific: in MON, the κ-CN-associated peak disappeared after fixing the upstream, missense, or 5′-UTR variant; in HOL, the peak disappeared after fixing the upstream, 3′-UTR, or downstream variant; but in NOR, the peak remained with the inclusion in the model of any of the four variants. Thus, none of the four candidate variants succeeded in explaining all the effects observed on κ-CN in the three breeds.

Instead, the peaks associated with the αs2-CN and β-CN phenotypes in NOR and the PC and αs2-CN phenotypes in MON could be explained by two distinct groups of six SNPs in complete LD, which were respectively located in the *CSN2* gene (three downstream and three intronic) and in the upstream region of the *ODAM* (odontogenic ameloblast-associated) gene (between the *CSN1S2* and *CSN3* genes).

Finally, the A1/B and A2 variants of *CSN2*, which ranked 147th and 86th, respectively, for their effects on PC and αs2-CN in NOR, were responsible for the αs2-CN phenotype in NOR but not for any other effect on the other traits or in the other breeds.

These results illustrate the complexity that is inherent with the analysis of the casein gene cluster, which contains the four genes *CSN1S1* **-** *CSN2* **-** *CSN1S2* **-** *CSN3* (encoding, respectively, αs1, β, αs2, and κ caseins). The polymorphisms of the amino-acid sequences of caseins are well known, and the effects on milk composition and cheese-making abilities have been well described (reviewed in Grosclaude et al. [34] and Caroli et al. [35]). Nevertheless, the effects of known polymorphisms are not always consistent between studies, likely because variations in the content of individual caseins are caused by several linked polymorphisms in the casein genes. Thus, it is likely that the most significant variants highlighted in our study are those that better explain haplotype effects. A multi-marker approach could facilitate efforts to distinguish the effects of all the causal polymorphisms located in this region.

### Pleiotropic effects of the *PAEP* gene region (BTA11)

The most significant effects on protein composition were found for variants that are located on BTA11. Contents of each individual protein in milk, with the exception of αs2-CN, were affected by this region in all three breeds. Effects were most significant for β-LG and, to a lesser extent, for κ-CN in all within-and multi-breed analyses. All of the most significant variants were located in or close to the *PAEP* (*progestagen*-*associated endometrial protein*) gene, also named *LGB* gene, which encodes the β-LG protein. The β-LG protein variants A and B, which are common in most cattle breeds, are associated with different β-LG levels in milk [34]. They differ by two amino-acid substitutions, caused by two missense mutations at 103,303,475 and 103,304,757 bp [36]. Interestingly, although these two variants had highly significant effects on β-LG in our study, they did not rank high in the peaks. In the MON and NOR analyses, both mutations were in complete LD and ranked 85th and 213rd, respectively, while in HOL, the two mutations ranked 48th and 109th, respectively (116th and 120th in multi-breed analysis). As suggested by Ganai et al. [36], differences in β-LG content may be caused by different levels of expression of the A and B alleles rather than by the direct effect of amino-acid substitutions. Among the top 30 variants in the within-and multi-breed analyses, only one, located at 103,298,431 bp in the upstream region of the *PAEP* gene, was shared by the four analyses. Moreover, this variant is one of the most significant in each analysis, ranking 6th, 4th, 1st, and 3rd, respectively, in the MON, NOR, HOL, and multi-breed analyses. The inclusion in conditional analyses of one of the causal missense variants or the most probable upstream variant identified in our study led to similar results in MON and HOL but not in NOR (see Additional file 6: Figure S1d). A peak remained in the conditional NOR analysis when missense mutations were fixed, but disappeared with the inclusion of the upstream variant at 103,298,431 bp. Thus, these results indicate that the missense mutations that cause the A and B variant protein polymorphisms do not explain all the variation associated with this region. Another variant, which is located in a regulatory region of *PAEP*, is more or less linked to the missense variants depending on the breed and appears to be a good candidate to explain different levels of expression of β-LG protein variants.

### Pleiotropic effects of the *DGAT1* gene region (BTA14)

Very significant effects on different protein composition traits were associated with the region of the *DGAT1* gene in NOR and HOL but not in MON. This region affected PC and κ-CN in both NOR and HOL; αs1-CN, β-CN, and α-LA only in NOR, and αs2-CN only in HOL. Moreover, individual proteins with the lowest *P* value were κ-CN in NOR and αs2-CN in HOL. The A allele of the *DGAT1* K232A polymorphism, which decreases fat and protein percentages as well as fat yield, and increases milk and protein yields [37], was present at a frequency of 9.4% in NOR, 15.8% in HOL, and only 0.6% in MON. However, our study confirmed that this causative variant was not the most significant for all traits analyzed. It ranked 18th to 72th among variants in the NOR analysis, depending on the trait, and outside the confidence interval for all traits in HOL. These results suggest, first, that not all variations observed in this region are associated with the K232A polymorphism and, second, that other specific causative mutations could explain the effects detected in NOR and HOL.

A large number of genes are annotated in the 1-Mbp region between 1.5 and 2.5 Mbp on BTA14 and, depending on the trait and the breed, between 66 and 494 variants located within the confidence intervals of this QTL are located in 17 to 30 of those genes. Among the top 50 variants for all traits, six were missense variants, of which two were found in NOR (*DGAT1* and *BOP1*) and four in HOL (three in *RECQL4* and one in *MROH1*). In this region, no variant remained significant in the conditional analyses for NOR when the *DGAT1* (K232A) or *BOP1* (1,842,678 bp) variants were included, and for HOL when the variants in *RECQL4* (one of the three variants in complete LD: 1,617,841, 1,618,978 and 1,619,555 bp) or *MROH1* (1,878,165 bp) were included. In contrast, a less significant peak persisted when the *DGAT1* or *BOP1* variant was included in the HOL analyses and when the *RECQL4* or *MROH1* variant was included in the NOR analyses (see Additional file 6: Figure S1e). Among the six missense variants, only the *RECQL4* variant at 1,617,841 bp has a predicted deleterious effect, with a SIFT score of zero. Therefore, in addition to the *DGAT1* K232A polymorphism previously identified as having effects on milk composition, we report additional candidate missense mutations in *BOP1*, *MROH1*, and *RECQL4* genes, which could be partly responsible for the effects associated with the centromeric end of BTA14.

### *ANKH* (BTA20) and α-LA

The GWAS on WGS data detected a QTL with very significant effects on the α-LA phenotype in all three within-breed analyses and in the multi-breed analysis; this confirmed our previous report based on a GWAS using 50 K SNP data [8]. Confidence intervals of the QTL included between one and four genes depending on the within- or multi-breed analysis, and *ANKH* was the only gene to be highlighted in all four analyses. *ANKH* encodes an inorganic pyrophosphate transport regulator that helps to prevent the deposition of minerals (calcium and phosphorous) in bones and α-LA exhibits a high affinity to metal ions, calcium in particular. In addition, *ANKH* is highly expressed in mammary tissue in Holstein and Jersey cows [27] and we observed a significant interaction between *ANKH* and *ALPL* (candidate gene on BTA2 for effects on αs2-CN), which suggests a functional link between these two genes (Fig. 4). Thus, *ANKH* constitutes a good functional candidate for effects on α-LA in HOL, MON, and NOR. However, none of the top 50 variants in this QTL were shared among the three breeds. In each breed, the most significant variant was located either in intronic regions of the *ANKH* gene (at 58,422,697 bp in NOR and at 58,450,656 bp in multi-breed analyses) or in an intergenic region. In MON and HOL, for which the most significant variants were intergenic, *ANKH* intronic variants ranked 2nd (at 58,446,560 bp) and 13th (at 58,491,204 bp), respectively. After fixing the most significant variant from each within-breed analysis, a peak remained in all conditional analyses (see Additional file 6: Figure S1f), which suggests that several causative mutations in the *ANKH* gene could be responsible for the variation of the amount of α-LA in milk. The most significant variants could be those that are most tightly linked to the causative mutations in each breed, which could explain why they were breed-specific.

### *AGPAT6* (BTA27) and κ-CN

The multi-breed analysis detected a QTL for κ-CN content located at about 36.2 Mbp on BTA27, while in within-breed analyses, peaks were present in MON and NOR but they did not reach significance (−log_10_(*P*) < 8.4), and no peak was observed in HOL (see Additional file 6: Figure S1g). In the multi-breed analysis, the four most significant variants were located in an intergenic region but the variants that ranked 5th to 17th were located in the *AGPAT6* gene, which was previously described as a functional gene for milk fat content with pleiotropic effects on other milk components, in particular protein content [38]. The five most significant variants in the gene were in complete LD and located in the upstream region (at 36,209,319, 36,211,252, 36,211,258, and 36,211,708 bp) or in the 5′-UTR region (at 36,212,352 bp) of the *AGPAT6* gene. For the five linked variants, MAF were equal to 0.46 in MON, 0.47 in NOR, and 0.39 in HOL (0.44 in multi-breed population). When the κ-CN phenotype was conditioned on the effect of any of these mutations, the association signals completely disappeared in the MON, NOR, and multi-breed analyses (see Additional file 6: Figure S1g). The four variants located in the upstream region were previously identified as candidate causal polymorphisms in both Holstein and Fleckvieh cows by Daetwyler et al. [9]. These authors pointed to the polymorphism at 36,211,252 bp as the most plausible causative mutation because it presented a high probability of being within a transcription binding site. In addition, Littlejohn et al. [38] described strong associations between milk composition traits (fat, protein, and lactose) and 10 variants in the *AGPAT6* gene. Three of these 10 variants were among the most significant variants in our study, located at 36,209,319, 36,211,708, and 36,212,352 bp. Thus, we identified five putative causative variants in the *AGPAT6* gene for milk protein composition; of these, the variant at 36,212,352 bp appears to be the most plausible causative mutation because it is located in the 5′-UTR region of the *AGPAT6* gene. However, the lack of a significant effect in the HOL analyses, in spite of the high MAF of the candidate variants, probably reflects additional effects yet to be explained.

### *PICALM* (BTA29) and αs1-CN

The αs1-CN phenotype was influenced by a genomic region that is located at about 9.5 Mbp on BTA29. Significant associations were found in MON, HOL, and multi-breed analyses, and a peak close to significance was found in NOR (−log_10_(*P*) = 7.9) (see Additional file 6: Figure S1h). In the MON and HOL analyses, the most significant variants were intergenic and, likewise, in the multi-breed analysis, all nine variants located within the confidence interval were intergenic. The most significant non-intergenic variants were located in the *PICALM* gene in MON and HOL. Two intronic variants ranked 11th in the peak detected in MON (9,651,065 and 9,656,439 bp) and one variant that ranked 10th in the HOL analysis, is located in the upstream region of the gene (9,611,304 bp). When conditional GWAS analyses were performed, the inclusion of the intronic variants removed the peak in MON but not in HOL analyses, and conversely, inclusion of the upstream variant removed the peak in HOL but not in MON analyses. In NOR, the peak in question persisted when either intronic or upstream variants were fixed (see Additional file 6: Figure S1h). These results suggest that either the causative variant is different between breeds or that several linked causative variants explain the significant effects observed in this region. The *PICALM* gene encodes a phosphatidylinositol-binding clathrin assembly protein, and polymorphisms in this gene are associated with the risk of Alzheimer’s disease [39] in humans. However, to date, no link was reported between polymorphisms in this gene and bovine milk composition

## Conclusions

Our study provides evidence that a GWAS-based approach applied to fine-scale phenotypes, whole-genome sequences, and multiple breeds provides enough resolution to identify candidate genes and directly pinpoint a limited number of candidate variants in most of these genes. Several variants, some shared among breeds, were identified as plausible candidate mutations for changes in milk protein composition in the three main French dairy cattle breeds. They were located both in genes that had previously been found to affect milk composition (*SLC37A1*, *MGST1*, *ABCG2*, *CSN1S1*, *CSN2*, *CSN1S2*, *CSN3*, *PAEP*, *DGAT1*, *AGPAT6*) and in genes for which no such relationship was known (*ALPL*, *ANKH*, *PICALM*). In the future, functional analyses will enable the establishment of causative links between these candidate variants and milk protein phenotypes. However, even before such studies are completed, our results offer the opportunity to improve cheese-making properties through the identification of genetic variants associated with changes in milk composition. Direct consequences of these results on practical selection are not obvious and depend on potential premiums on protein composition and on incentives proposed by the milk processing industry. Nevertheless, it would be desirable to favour caseins against whey proteins at least for milk collected for cheese production. Such an option could be implemented by including variants that affect individual proteins in genomic evaluation models.

## Additional files

**Additional file 1: Table S1.** The 1000 bull genome population (RUN4). (Daetwyler HD, personal communication).

**Additional file 2: Table S2.** Number of variants included within confidence intervals for each QTL region and trait, regardless of breed.

**Additional file 3: Table S3.** Description of the pleiotropic QTL regions detected in within-breed (MON, NOR, or HOL) or multi-breed (Multi) analyses.

**Additional file 4: Table S4.** Description of other significant QTL regions detected in within-breed (MON, NOR, or HOL) or multi-breed (Multi) analyses.

**Additional file 5: Table S5.** Functional annotations of variants included within confidence intervals (± 100 kb) of the 34 QTL for each trait in the three within-breed Montbéliarde (MON), Normande (NOR), and Holstein (HOL) or in multi-breed analyses.

**Additional file 6: Figure S1.** −log_10_(*P*) plotted against the position of variants detected by GWAS (in *grey*) and conditional GWAS (GWAS_COJO; in *blue*) **a** On BTA1, **b** BTA2, **c** BTA5, **d** BTA11, **e** BTA14, **f** BTA20, **g** BTA27 and **h** BTA29
