# Supplementary material for: Within-breed and multi-breed GWAS on imputed whole-genome sequence variants reveal candidate mutations affecting milk protein composition in dairy cattle
Source: Genet Sel Evol. 2017 Sep 18;49:68. doi: 10.1186/s12711-017-0344-z (PMC5604355; doi:10.1186/s12711-017-0344-z)
Supplement: Supplementary file 1 — Additional file 1: Table S1. The 1000 bull genome population (RUN4). (Daetwyler HD, personal communication). [file 12711_2017_344_MOESM1_ESM.docx]

**Table S1.** The 1000 bull genome population (Run4: Daetwyler HD, personal communication)

| **Breed / Cross** | **Number** |
| --- | --- |
| **Holstein (Black and White)** | **288** |
| Simmental (Dual and Beef) | 216 |
| Angus (Black and Red) | 138 |
| Jersey | 61 |
| Brown Swiss | 59 |
| Gelbvieh | 34 |
| Charolais | 33 |
| Hereford | 31 |
| Limousin | 31 |
| Guelph Composite | 30 |
| Beef Booster | 29 |
| Alberta Composite | 28 |
| **Montbeliarde** | **28** |
| Ayrshire Finnish | 25 |
| **Normande** | **24** |
| Holstein (Red and White) | 23 |
| Swedish Red | 16 |
| Danish Red | 15 |
| Other crosses | 11 |
| Belgian Blue | 10 |
| Piedmontese | 5 |
| Eringer | 2 |
| Galloway | 2 |
| Unknown | 2 |
| Scottish Highland | 2 |
| Pezzata Rossa Italiana | 1 |
| Romagnola | 1 |
| Salers | 1 |
| Tyrolean Grey | 1 |
| **Total** | **1147** |
