# Supplementary material for: Within-breed and multi-breed GWAS on imputed whole-genome sequence variants reveal candidate mutations affecting milk protein composition in dairy cattle
Source: Genet Sel Evol. 2017 Sep 18;49:68. doi: 10.1186/s12711-017-0344-z (PMC5604355; doi:10.1186/s12711-017-0344-z)
Supplement: Supplementary file 2 — Additional file 2: Table S2. Number of variants included within confidence intervals for each QTL region and trait, regardless of breed. [file 12711_2017_344_MOESM2_ESM.docx]

**Table S2.** Number of variants included within confidence intervals for each QTL region and trait, regardless of breed

| QTL region | BTA | PC | α-LA | β-LG | Σ-WP | α_s1_-CN | α_s2_-CN | β-CN | κ-CN | Σ-CN | # distinct variants |
| --- | --- | --- | --- | --- | --- | --- | --- | --- | --- | --- | --- |
| 1 | 1 | 0 | 86 | 0 | 0 | 324 | 0 | 0 | 0 | 0 | 329 |
| 2 | 2 | 0 | 0 | 0 | 0 | 311 | 0 | 0 | 0 | 0 | 311 |
| 3 | 2 | 0 | 0 | 0 | 0 | 0 | 106 | 83 | 105 | 0 | 124 |
| 4 | 2 | 0 | 0 | 0 | 0 | 0 | 4 | 0 | 0 | 0 | 4 |
| 5 | 2 | 0 | 0 | 0 | 0 | 0 | 5 | 0 | 0 | 0 | 5 |
| 6 | 3 | 23 | 0 | 0 | 0 | 0 | 113 | 0 | 0 | 0 | 133 |
| 7 | 4 | 0 | 0 | 0 | 0 | 0 | 12 | 17 | 0 | 0 | 17 |
| 8 | 5 | 63 | 0 | 0 | 0 | 0 | 0 | 0 | 0 | 0 | 63 |
| 9 | 5 | 0 | 0 | 0 | 0 | 36 | 0 | 0 | 0 | 0 | 36 |
| 10 | 6 | 0 | 0 | 0 | 0 | 118 | 0 | 0 | 0 | 0 | 118 |
| 11 | 6 | 0 | 0 | 0 | 0 | 40 | 0 | 0 | 0 | 0 | 40 |
| 12 | 6 | 0 | 0 | 0 | 0 | 324 | 0 | 0 | 0 | 0 | 324 |
| 13 | 6 | 0 | 0 | 0 | 0 | 85 | 0 | 0 | 0 | 0 | 85 |
| 14 | 6 | 0 | 0 | 0 | 0 | 9 | 0 | 0 | 0 | 0 | 9 |
| 15 | 6 | 0 | 0 | 0 | 0 | 0 | 11 | 0 | 0 | 0 | 11 |
| 16 | 6 | 868 | 0 | 400 | 0 | 429 | 592 | 1,099 | 1,023 | 0 | 2,197 |
| 17 | 10 | 0 | 0 | 0 | 0 | 0 | 0 | 123 | 0 | 0 | 123 |
| 18 | 11 | 0 | 337 | 312 | 349 | 378 | 271 | 356 | 313 | 315 | 498 |
| 19 | 14 | 745 | 332 | 0 | 0 | 239 | 316 | 335 | 570 | 0 | 765 |
| 20 | 14 | 52 | 0 | 0 | 0 | 0 | 0 | 0 | 0 | 0 | 52 |
| 21 | 14 | 4 | 0 | 0 | 0 | 0 | 0 | 0 | 0 | 0 | 4 |
| 22 | 16 | 0 | 0 | 0 | 0 | 0 | 0 | 0 | 3 | 0 | 3 |
| 23 | 17 | 0 | 0 | 0 | 0 | 0 | 0 | 57 | 0 | 0 | 57 |
| 24 | 19 | 0 | 0 | 0 | 0 | 161 | 0 | 0 | 0 | 0 | 161 |
| 25 | 20 | 28 | 0 | 0 | 0 | 0 | 0 | 0 | 0 | 0 | 28 |
| 26 | 20 | 279 | 0 | 0 | 0 | 0 | 0 | 0 | 0 | 0 | 279 |
| 27 | 20 | 0 | 78 | 0 | 0 | 0 | 0 | 0 | 0 | 0 | 78 |
| 28 | 20 | 0 | 1,536 | 0 | 0 | 0 | 17 | 0 | 0 | 0 | 1,538 |
| 29 | 21 | 0 | 0 | 0 | 0 | 0 | 0 | 84 | 0 | 0 | 84 |
| 30 | 22 | 0 | 222 | 0 | 0 | 0 | 0 | 0 | 0 | 0 | 222 |
| 31 | 25 | 0 | 0 | 0 | 0 | 0 | 0 | 0 | 0 | 203 | 203 |
| 32 | 27 | 0 | 0 | 0 | 0 | 0 | 0 | 0 | 25 | 0 | 25 |
| 33 | 29 | 0 | 0 | 0 | 0 | 228 | 0 | 0 | 0 | 0 | 228 |
| 34 | 29 | 83 | 0 | 0 | 0 | 0 | 0 | 0 | 0 | 0 | 83 |
| Total |  | 2,145 | 2,591 | 712 | 349 | 2,682 | 1,447 | 2,154 | 2,039 | 518 | 8,237 |
