# Supplementary material for: Within-breed and multi-breed GWAS on imputed whole-genome sequence variants reveal candidate mutations affecting milk protein composition in dairy cattle
Source: Genet Sel Evol. 2017 Sep 18;49:68. doi: 10.1186/s12711-017-0344-z (PMC5604355; doi:10.1186/s12711-017-0344-z)
Supplement: Supplementary file 3 — Additional file 3: Table S3. Description of the pleiotropic QTL regions detected in within-breed (MON, NOR, or HOL) or multi-breed (Multi) analyses. [file 12711_2017_344_MOESM3_ESM.docx]

**Table S3** Description of the pleiotropic QTL regions detected in within-breed (MON, NOR, or HOL) or multi-breed (Multi) analyses

| BTA | QTL | Breed | Trait | Start (bp) | End (bp) | Nb significant. variants | Nb significant variants in genes | Nb genes | Top variant positions (bp)^a^ | -log10(P) max | MAF | b | SE | Top variant gene | Top genic variant gene | Top/top genic variant annotation (Nb of variants) |
| --- | --- | --- | --- | --- | --- | --- | --- | --- | --- | --- | --- | --- | --- | --- | --- | --- |
| 6 | 16 | MON | PC | 86659882 | 87332009 | 156 | 152 | 3 | **87319671-87324778** | 21.0 | 0.37 | -0.06 | 0.006 | *ODAM* | *ODAM* | up. (4) |
| 6 | 16 | MON | αs1-CN | 85269591 | 85322964 | 39 | 39 | 1 | 85273469 | 9.8 | 0.36 | -0.05 | 0.008 | *TMPRSS11D* | *TMPRSS11D* | intron |
| 6 | 16 | MON | αs2-CN | 86652247 | 87685742 | 85 | 78 | 4 | **87319671-87324778** | 16.5 | 0.37 | -0.04 | 0.004 | *ODAM* | *ODAM* | up. (4) |
| 6 | 16 | MON | β-LG | 87333049 | 88217982 | 192 | 89 | 8 | 87903002 | 12.3 | 0.27 | 0.17 | 0.023 | *RUFY3* | *RUFY3* | intron |
| 6 | 16 | MON | β-CN | 84488163 | 85395384 | 138 | 29 | 3 | 85203258-85206608 | 12.4 | 0.41 | -0.10 | 0.013 | *38648*^b^ | *38648*^b^ | intron (17) |
| 6 | 16 | MON | κ-CN | 86609381 | 87422461 | 228 | 123 | 8 | 87407150 | 23.9 | 0.48 | 0.08 | 0.008 | *-* | *CSN3* | 3 ‘ UTR |
| 6 | 16 | NOR | PC | 86569668 | 87304763 | 150 | 61 | 6 | **87168455** | 17.2 | 0.47 | 0.07 | 0.008 | *-* | *CSN1S2* | Intron |
| 6 | 16 | NOR | αs2-CN | 86569668 | 87848848 | 114 | 41 | 7 | 87168181-87191866 | 11.4 | 0.47 | 0.03 | 0.004 | *CSN2/HSTN* | *CSN2/HSTN* | CSN2 (3 down. + 3 intron) HSTN (2 intron) |
| 6 | 16 | NOR | β-CN | 86569668 | 87882986 | 196 | 67 | 10 | **87168455** | 10.7 | 0.47 | -0.12 | 0.018 | *-* | *CSN2* | down. (3) intron (3) |
| 6 | 16 | NOR | κ-CN | 86558890 | 88101922 | 872 | 450 | 8 | **87376747** | 22.1 | 0.11 | 0.14 | 0.015 | *CSN3* | *CSN3* | up. |
| 6 | 16 | HOL | PC | 86992137 | 88058629 | 565 | 334 | 10 | 87062878 | 10.0 | 0.15 | 0.07 | 0.011 | *SULT1E1* | *SULT1E1* | intron |
| 6 | 16 | HOL | αs1-CN | 87318422 | 88262925 | 388 | 110 | 3 | 87424477 | 11.6 | 0.35 | 0.07 | 0.010 | *-* | *CSN3* | intron |
| 6 | 16 | HOL | αs2-CN | 86992137 | 88116372 | 402 | 238 | 7 | **87154594** | 8.7 | 0.20 | -0.04 | 0.007 | *CSN1S1* | *CSN1S1* | intron |
| 6 | 16 | HOL | β-CN | 87062878 | 87428475 | 768 | 334 | 5 | 87402422 | 9.6 | 0.22 | 0.13 | 0.021 | *-* | *CSN3* | up. (1) intron (2) |
| 6 | 16 | HOL | κ-CN | 87355621 | 87428475 | 389 | 107 | 1 | 87407145 | 45.5 | 0.38 | 0.16 | 0.011 | *-* | *CSN3* | down. |
| 6 | 16 | multi | PC | 87154594 | 87155863 | 2 | 2 | 1 | **87154594** | 36.1 | 0.33 | -0.06 | 0.004 | *CSN1S1* | *CSN1S1* | intron |
| 6 | 16 | multi | αs1-CN | 87332537 | 87422713 | 342 | 102 | 2 | 87403760 | 18.3 | 0.44 | -0.05 | 0.005 | *-* | *CSN3* | down. |
| 6 | 16 | multi | αs2-CN | 87154594 | 87155863 | 2 | 2 | 1 | **87154594** | 27.2 | 0.33 | -0.03 | 0.003 | *CSN1S1* | *CSN1S1* | intron |
| 6 | 16 | multi | β-LG | 87167953 | 88238729 | 216 | 104 | 7 | **87376747** | 12.2 | 0.42 | -0.11 | 0.015 | *CSN3* | *CSN3* | up. |
| 6 | 16 | multi | β-CN | 87154594 | 87407150 | 145 | 91 | 4 | **87154594** | 18.7 | 0.33 | 0.09 | 0.010 | *CSN1S1* | *CSN1S1* | intron |
| 6 | 16 | multi | κ-CN | 87355621 | 87426173 | 374 | 108 | 1 | 87392592 | 71.2 | 0.44 | 0.12 | 0.006 | *CSN3* | *CSN3* | 3 ‘ UTR |
| 11 | 18 | MON | αs1-CN | 103050473 | 103386323 | 335 | 235 | 6 | 103293198 | 19.6 | 0.49 | 0.06 | 0.007 | *-* | *PAEP* | up. |
| 11 | 18 | MON | α-LA | 103270401 | 103386323 | 311 | 196 | 3 | 103380040 | 12.2 | 0.41 | 0.03 | 0.004 | *-* | *PAEP* | up. |
| 11 | 18 | MON | β-LG | 103270401 | 103322890 | 262 | 204 | 3 | **103289035-103308330** | 278.7 | 0.49 | -0.64 | 0.018 | *PAEP/48091*^b^ | *PAEP/48091*^b^ | PAEP (2 intron + 1 down.) ND (1 down.) |
| 11 | 18 | MON | β−CN | 103050134 | 104237602 | 268 | 208 | 5 | 103299937 | 15.2 | 0.49 | 0.09 | 0.011 | *PAEP* | *PAEP* | up. |
| 11 | 18 | MON | κ-CN | 103273963 | 103322890 | 262 | 204 | 3 | 103293584 | 57.3 | 0.49 | 0.12 | 0.007 | *-* | *48091*^b^ | intron (2) |
| 11 | 18 | MON | Σ-WP | 103270401 | 103322890 | 262 | 204 | 3 | **103289035-103308330** | 202.1 | 0.49 | -0.46 | 0.015 | *48091*^b^ | *48091*^b^ | down. |
| 11 | 18 | MON | Σ-CN | 103270401 | 103322890 | 262 | 204 | 3 | 103299858 | 154.4 | 0.49 | 0.29 | 0.011 | *PAEP* | *PAEP* | up. |
| 11 | 18 | NOR | αs1-CN | 103273963 | 103356124 | 294 | 233 | 4 | 103301443-**103301805** | 20.5 | 0.42 | 0.06 | 0.006 | *PAEP* | *PAEP* | PAEP (2 up. + 2 5’ UTR + 2 syn. + 1 intron) |
| 11 | 18 | NOR | β-LG | 103275740 | 103362003 | 304 | 241 | 4 | **103301202** | 254.8 | 0.42 | -0.4 | 0.019 | *PAEP* | *PAEP* | up. |
| 11 | 18 | NOR | β-CN | 103285442 | 103361480 | 259 | 212 | 4 | 103296920 | 9.9 | 0.42 | 0.09 | 0.014 | *PAEP* | *PAEP* | up. |
| 11 | 18 | NOR | κ-CN | 103275740 | 103362003 | 304 | 241 | 4 | **103301202** | 50.9 | 0.42 | 0.12 | 0.008 | *PAEP* | *PAEP* | up. |
| 11 | 18 | NOR | Σ-WP | 103275740 | 103362003 | 304 | 241 | 4 | **103301202** | 205.1 | 0.42 | -0.49 | 0.016 | *PAEP* | *PAEP* | up. |
| 11 | 18 | NOR | Σ-CN | 103275740 | 103362003 | 304 | 241 | 4 | **103301202** | 136.2 | 0.42 | 0.28 | 0.011 | *PAEP* | *PAEP* | up. |
| 11 | 18 | HOL | αs1-CN | 103275740 | 103326758 | 245 | 198 | 4 | **103301805** | 9.7 | 0.47 | 0.05 | 0.008 | *PAEP* | *PAEP* | intron |
| 11 | 18 | HOL | β-LG | 103286203 | 103322890 | 231 | 185 | 4 | **103297793-103301690** | 225.9 | 0.47 | -0.73 | 0.023 | *PAEP* | *PAEP* | PAEP (17 up. + 1 5 ‘ UTR) |
| 11 | 18 | HOL | β-CN | 103291134 | 103332699 | 208 | 162 | 4 | 103295526 | 16.8 | 0.47 | 0.12 | 0.014 | *-* | *48091*^b^ | down. |
| 11 | 18 | HOL | κ-CN | 103017641 | 103322890 | 231 | 186 | 5 | 103291134/103291146 | 47.0 | 0.47 | 0.14 | 0.010 | *48091*^b^ | *48091*^b^ | down. (2) |
| 11 | 18 | HOL | Σ-WP | 103276931 | 103322890 | 278 | 219 | 4 | **103297793-103301690** | 206.0 | 0.47 | -0.58 | 0.019 | *PAEP* | *PAEP* | up. (17) 5’ UTR (1) |
| 11 | 18 | HOL | Σ-CN | 103279969 | 103322890 | 232 | 186 | 4 | 103301694-103308459 | 143.5 | 0.47 | 0.34 | 0.013 | *PAEP* | *PAEP* | 5’ UTR (1) syn. (1) down. (1) |
| 11 | 18 | multi | αs1-CN | 103276931 | 103322890 | 268 | 210 | 4 | 103301805 | 43.9 | 0.49 | 0.06 | 0.004 | *PAEP* | *PAEP* | intron |
| 11 | 18 | multi | αs2-CN | 103275439 | 103356124 | 271 | 210 | 4 | 103288459 | 15.9 | 0.45 | 0.02 | 0.002 | *48091*^b^ | *48091* | down. |
| 11 | 18 | multi | α-LA | 103275740 | 103322890 | 270 | 211 | 4 | 103291319 | 21.6 | 0.47 | 0.03 | 0.003 | *48091*^b^ | *48091*^b^ | down. (3) |
| 11 | 18 | multi | β-LG | 103275740 | 103322890 | 276 | 216 | 4 | 103302351 | 612.4 | 0.49 | -0.66 | 0.011 | *PAEP* | *PAEP* | intron |
| 11 | 18 | multi | β-CN | 103276931 | 103322890 | 267 | 214 | 4 | 103300672 | 36.4 | 0.49 | 0.10 | 0.008 | *PAEP* | *PAEP* | up. |
| 11 | 18 | multi | κ-CN | 103276931 | 103322890 | 273 | 215 | 4 | 103300719/103301063 | 148.6 | 0.49 | 0.13 | 0.005 | *PAEP* | *PAEP* | up. (2) |
| 11 | 18 | multi | Σ-WP | 103273963 | 103361645 | 55 | 49 | 3 | 103273963 | 304.7 | 0.48 | -0.34 | 0.009 | *-* | *PAEP* | up. |
| 11 | 18 | multi | Σ-CN | 103273963 | 103356124 | 34 | 25 | 4 | 103277725 | 304.1 | 0.48 | 0.25 | 0.007 | *-* | *48091*^b^ | up. |
| 14 | 19 | NOR | PC | 1431996 | 2555954 | 494 | 502 | 32 | **1828456-1855090** | 17.1 | 0.09 | -0.09 | 0.010 | *BOP1/MROH1* | *BOP1/MROH1* | 9 BOP1 (7 intron + 1 syn. + 1 missense) 1 MROH1 (intron) |
| 14 | 19 | NOR | αs1-CN | 1704594 | 1965628 | 178 | 169 | 17 | 1843574 | 9.7 | 0.09 | -0.06 | 0.010 | *BOP1* | *BOP1* | intron |
| 14 | 19 | NOR | α-LA | 1550766 | 2517005 | 314 | 299 | 22 | **1857348-1892559** | 12.9 | 0.09 | -0.06 | 0.008 | *MROH1* | *MROH1* | intron (15) Syn. (2) |
| 14 | 19 | NOR | β-CN | 1550766 | 2517005 | 335 | 317 | 23 | **1857348-1892559** | 15.5 | 0.09 | 0.19 | 0.023 | *MROH1* | *MROH1* | intron (15) Syn. (2) |
| 14 | 19 | NOR | κ-CN | 1550766 | 2527051 | 317 | 302 | 22 | **1828456-1855090** | 19.7 | 0.09 | -0.12 | 0.013 | *BOP1/MROH1* | *BOP1/MROH1* | 9 BOP1 (7 intron + 1 syn. + 1 missense) 1 MROH1 (intron) |
| 14 | 19 | HOL | PC | 1569809 | 2146964 | 311 | 259 | 29 | 1681353 | 36.6 | 0.21 | -0.10 | 0.008 | *TONSL* | *TONSL* | up. |
| 14 | 19 | HOL | αs2-CN | 1569809 | 2052641 | 316 | 264 | 30 | 1806875-1831642 | 23.0 | 0.21 | 0.06 | 0.006 | *HSF1/BOP1* | *HSF1/BOP1* | 7 HSF1 (1 syn. + 5 intron + 1 up.) 1 BOP1 (intron) |
| 14 | 19 | HOL | κ-CN | 1569809 | 2146964 | 313 | 260 | 29 | 1699016 | 15.5 | 0.21 | -0.09 | 0.011 | *VPS28* | *VPS28* | down. |
| 14 | 19 | multi | PC | 1717081 | 1892784 | 111 | 111 | 12 | **1816568** | 58.1 | 0.09 | -0.09 | 0.006 | *HSF1* | *HSF1* | intron |
| 14 | 19 | multi | αs1-CN | 1717081 | 1892559 | 141 | 141 | 12 | 1759054-1765055 | 10.1 | 0.09 | -0.04 | 0.007 | *GPR172B/TMEM249* | *GPR172B/TMEM249* | 9 GPR172B (8 down. + 1 missense) 1 TMEM249 (up.) |
| 14 | 19 | multi | αs2-CN | 1702310 | 2052641 | 153 | 122 | 22 | 1929994 | 27.5 | 0.06 | 0.05 | 0.005 | *SHARPIN* | *SHARPIN* | down. |
| 14 | 19 | multi | α-LA | 1550766 | 2045363 | 114 | 106 | 20 | 2044108 | 10.9 | 0.04 | -0.05 | 0.007 | *-* | *GPR172B* | down. (8) missense (1) |
| 14 | 19 | multi | β-CN | 1706207 | 1892559 | 73 | 66 | 11 | 1717081-1722853 | 23.8 | 0.10 | -0.13 | 0.012 | *SLC39A4* | *SLC39A4* | up. (1) intron (4) syn. (1) |
| 14 | 19 | multi | κ-CN | 1706207 | 1892559 | 136 | 130 | 12 | **1816568** | 37.1 | 0.09 | -0.10 | 0.008 | *HSF1* | *HSF1* | intron |

^a^Positions of the variants with the maximum –log_10_(*P*) or bounds of the interval, separated by a hyphen, that contained all the top variants if more than two variants were in the top

^b^xxxxx for ENSBTAG00000xxxxx

SE = standard error
