# Supplementary material for: Within-breed and multi-breed GWAS on imputed whole-genome sequence variants reveal candidate mutations affecting milk protein composition in dairy cattle
Source: Genet Sel Evol. 2017 Sep 18;49:68. doi: 10.1186/s12711-017-0344-z (PMC5604355; doi:10.1186/s12711-017-0344-z)
Supplement: Supplementary file 4 — Additional file 4: Table S4. Description of other significant QTL regions detected in within-breed (MON, NOR, or HOL) or multi-breed (Multi) analyses. [file 12711_2017_344_MOESM4_ESM.docx]

**Table S4** Description of other significant QTL regions detected in within-breed (MON, NOR, or HOL) or multi-breed (Multi) analyses

| BTA | QTL | Breed | Trait | Start (bp) | End (bp) | Nb signficant variants | Nb significant variants in genes | Nb genes | Top variant positions (bp)^a^ | -log10(P) max | MAF | b | SE | Top variant gene | Top genic variant gene | Top / top genic variant annotation (number of variants) |
| --- | --- | --- | --- | --- | --- | --- | --- | --- | --- | --- | --- | --- | --- | --- | --- | --- |
| 1 | 1 | MON | αs1-CN | 144213338 | 144464093 | 110 | 81 | 1 | 144397274 | 8.6 | 0.44 | -0.05 | 0.008 | *SLC37A1* | *SLC37A1* | intron |
| 1 | 1 | multi | αs1-CN | 144213338 | 144487373 | 298 | 138 | 1 | 144398814 | 12.8 | 0.49 | -0.03 | 0.005 | *SLC37A1* | *SLC37A1* | intron |
| 1 | 1 | multi | α-LA | 144207132 | 144470988 | 86 | 80 | 1 | 144401795 | 10.7 | 0.48 | 0.02 | 0.003 | *SLC37A1* | *SLC37A1* | intron |
| 2 | 2 | MON | αs1-CN | 5856554 | 7755903 | 311 | 114 | 9 | 6838378 | 11.1 | 0.15 | 0.07 | 0.010 | *-* | *OSGEPL1* | intron |
| 2 | 2 | multi | αs1-CN | 5788801 | 7179149 | 90 | 19 | 5 | 6379048 | 10.5 | 0.09 | -0.05 | 0.007 | *-* | *PMS1* | down. (1) intron (5) |
| 2 | 3 | NOR | αs2-CN | 131792733 | 132596006 | 61 | 61 | 4 | **131806882** | 12.0 | 0.37 | -0.03 | 0.004 | *ALPL* | *ALPL* | intron |
| 2 | 3 | multi | αs2-CN | 131792733 | 131888417 | 97 | 92 | 2 | **131806882** | 20.8 | 0.50 | -0.02 | 0.003 | *ALPL* | *ALPL* | intron |
| 2 | 3 | multi | β-CN | 131711660 | 131888417 | 83 | 75 | 3 | **131809031** | 14.9 | 0.31 | 0.07 | 0.009 | *ALPL* | *ALPL* | intron |
| 2 | 3 | multi | κ-CN | 131711660 | 131850456 | 105 | 105 | 3 | **131809031** | 8.8 | 0.1 | -0.04 | 0.006 | *ALPL* | *ALPL* | intron |
| 2 | 4 | multi | αs2-CN | 134223357 | 134270994 | 4 | 0 | 0 | 134223740 | 10.1 | 0.09 | -0.02 | 0.004 | *-* | *-* | intron |
| 2 | 5 | NOR | αs2-CN | 136085706 | 136108115 | 5 | 5 | 1 | 136091182 | 8.4 | 0.23 | 0.02 | 0.004 | *PADI2* | *PADI2* | intron |
| 3 | 6 | NOR | αs2-CN | 14787169 | 15555081 | 113 | 73 | 13 | 14787169-14799136 | 9.6 | 0.11 | 0.04 | 0.006 | *ND* | *ND* | up. (6) non-coding exon (1) down. (5) intergenic) |
| 3 | 6 | multi | PC | 14787510 | 15555081 | 23 | 19 | 3 | 15379528/15379666 | 9.9 | 0.23 | -0.03 | 0.005 | *FDPS* | *FDPS* | up. (2) |
| 4 | 7 | multi | αs2-CN | 77821164 | 77914524 | 12 | 12 | 4 | 77827342 | 9.2 | 0.05 | -0.03 | 0.005 | *GCK* | *GCK* | intron |
| 4 | 7 | multi | β-CN | 77668701 | 78844619 | 17 | 17 | 7 | 77869652 | 9.8 | 0.05 | 0.11 | 0.017 | *POLD2* | *POLD2* | up. |
| 5 | 8 | multi | PC | 93891948 | 94575375 | 63 | 51 | 1 | 93950211 | 9.3 | 0.22 | 0.03 | 0.005 | *MGST1* | *MGST1* | up. |
| 5 | 9 | MON | αs1-CN | 117224480 | 118540359 | 36 | 33 | 4 | 118127195 | 9.7 | 0.03 | -0.11 | 0.017 | *TBC1D22A* | *TBC1D22A* | intron |
| 6 | 10 | HOL | αs1-CN | 37596669 | 38368227 | 9 | 6 | 4 | 38015146/38020110 | 19.9 | 0.02 | 0.25 | 0.026 | *ABCG2* | *ABCG2* | intron (2) |
| 6 | 10 | multi | αs1-CN | 37571055 | 38368227 | 113 | 95 | 4 | 37596583 | 15.0 | 0.02 | -0.11 | 0.014 | *HERC3* | *HERC3* | intron |
| 6 | 11 | HOL | αs1-CN | 39947470 | 41267455 | 36 | 23 | 1 | 40267834 | 11.7 | 0.03 | -0.16 | 0.023 | *-* | *SLIT2* | intron (13) |
| 6 | 11 | multi | αs1-CN | 40269767 | 40898660 | 4 | 0 | 0 | 40269767 | 10.5 | 0.02 | -0.10 | 0.016 | *-* | *-* | - |
| 6 | 12 | MON | αs1-CN | 46568761 | 47052793 | 74 | 13 | 4 | 46874151 | 18.5 | 0.20 | -0.08 | 0.009 | *SEL1L3* | *SEL1L3* | intron |
| 6 | 12 | NOR | αs1-CN | 45835012 | 46629824 | 229 | 56 | 5 | 46604159 | 12.2 | 0.32 | 0.05 | 0.007 | *-* | *SEPSECS* | intron |
| 6 | 12 | HOL | αs1-CN | 45496362 | 45804827 | 78 | 23 | 1 | 45804771 | 9.4 | 0.03 | -0.14 | 0.022 | *-* | *DHX15* | intron (2) |
| 6 | 12 | multi | αs1-CN | 46452533 | 47009652 | 108 | 10 | 3 | 46610035 | 20.1 | 0.30 | 0.05 | 0.005 | *-* | *SEL1L3* | intron |
| 6 | 13 | MON | αs1-CN | 49757707 | 50680915 | 82 | 0 | 0 | **50661697** | 12.2 | 0.22 | 0.06 | 0.009 | *-* | *-* | - |
| 6 | 13 | multi | αs1-CN | 49721574 | 50680915 | 9 | 0 | 0 | **50661697** | 10.6 | 0.09 | 0.06 | 0.009 | *-* | *-* | - |
| 6 | 14 | multi | αs1-CN | 51814433 | 51943070 | 9 | 9 | 1 | 51850292 | 9.2 | 0.06 | -0.05 | 0.009 | *PCDH7* | *PCDH7* | intron |
| 6 | 15 | multi | αs2-CN | 61365935 | 61367705 | 11 | 11 | 1 | 61367193 | 8.8 | 0.26 | 0.02 | 0.003 | *APBB2* | *APBB2* | intron |
| 10 | 17 | multi | β-CN | 2062273 | 2140383 | 123 | 25 | 1 | 2135500 | 9.5 | 0.25 | 0.06 | 0.009 | *-* | *47622*^b^ | down. |
| 14 | 20 | HOL | PC | 65384515 | 67315582 | 45 | 31 | 6 | 66363500 | 8.5 | 0.06 | -0.08 | 0.014 | *SPAG1* | *SPAG1* | intron |
| 14 | 20 | multi | PC | 67569135 | 68074640 | 7 | 0 | 0 | 68074640 | 9.9 | 0.03 | 0.07 | 0.011 | *-* | *-* | - |
| 14 | 21 | multi | PC | 70397845 | 70422295 | 4 | 3 | 1 | 70397845-70406169 | 8.4 | 0.02 | 0.07 | 0.011 | *GDF6* | *GDF6* | up. (1) intron (2) |
| 16 | 22 | multi | κ-CN | 1603186 | 1609129 | 3 | 0 | 0 | 1608132 | 9.1 | 0.28 | -0.03 | 0.006 | *-* | *-* | - |
| 17 | 23 | multi | β-CN | 53027601 | 53282123 | 57 | 46 | 4 | 53051338 | 9.7 | 0.05 | -0.11 | 0.018 | *-* | *BRI3BP* | intron |
| 19 | 24 | MON | αs1-CN | 60419888 | 61404020 | 98 | 0 | 0 | 61396453 | 10.0 | 0.43 | 0.04 | 0.006 | *-* | *-* | - |
| 19 | 24 | multi | αs1-CN | 60991991 | 61135270 | 135 | 0 | 0 | 61134515 | 11.5 | 0.20 | -0.03 | 0.005 | *-* | *-* | - |
| 20 | 25 | multi | PC | 31201346 | 31606682 | 28 | 26 | 3 | 31346200 | 10.4 | 0.11 | 0.04 | 0.007 | *C20H5orf34* | *C20H5orf34* | intron |
| 20 | 26 | HOL | PC | 33264871 | 34278663 | 278 | 162 | 7 | 33775868 | 9.5 | 0.12 | 0.07 | 0.011 | *PTGER4* | *PTGER4* | up. |
| 20 | 26 | multi | PC | 33327244 | 34214514 | 48 | 20 | 2 | 34115618 | 9.8 | 0.05 | 0.05 | 0.009 | *-* | *MROH2B* | intron |
| 20 | 27 | MON | α-LA | 43644234 | 44802655 | 78 | 0 | 0 | 44036476 | 9.2 | 0.08 | 0.04 | 0.007 | *-* | *-* | - |
| 20 | 28 | MON | α-LA | 58245039 | 58457768 | 85 | 61 | 1 | 58287384 | 63.6 | 0.04 | 0.15 | 0.009 | *-* | *ANKH* | intron (5) |
| 20 | 28 | NOR | α-LA | 57500764 | 59210001 | 308 | 228 | 2 | 58422697/58422881 | 34.9 | 0.45 | 0.07 | 0.006 | *ANKH* | *ANKH* | intron (2) |
| 20 | 28 | HOL | α-LA | 57531993 | 58986739 | 1236 | 646 | 4 | 58256671 | 31.3 | 0.22 | 0.09 | 0.007 | *-* | *45869*^b^ | down. |
| 20 | 28 | multi | αs2-CN | 58214329 | 58281189 | 17 | 0 | 0 | 58258612 | 8.8 | 0.20 | -0.02 | 0.003 | *-* | *-* | - |
| 20 | 28 | multi | α-LA | 58256671 | 58472026 | 69 | 64 | 1 | 58450656 | 89.7 | 0.27 | 0.08 | 0.004 | *ANKH* | *ANKH* | intron |
| 21 | 29 | multi | β-CN | 37741448 | 37843274 | 84 | 0 | 0 | 37787544 | 8.5 | 0.06 | -0.10 | 0.017 | *-* | *-* | - |
| 22 | 30 | multi | α-LA | 54410659 | 55273619 | 222 | 213 | 2 | 54417572 | 8.8 | 0.28 | -0.02 | 0.003 | *LARS2* | *LARS2* | intron |
| 25 | 31 | HOL | Σ-CN | 26209288 | 26473010 | 203 | 167 | 12 | **26308666** | 15.9 | 0.23 | -0.14 | 0.017 | *1800* ^b^ | *18000*^b^ | intron |
| 25 | 31 | multi | Σ-CN | 26306271 | 26422946 | 166 | 150 | 5 | **26308666** | 19.3 | 0.09 | -0.12 | 0.013 | *18000*^b^ | *18000*^b^ | intron |
| 27 | 32 | multi | κ-CN | 36190361 | 36274468 | 25 | 15 | 2 | 36203904 | 11.6 | 0.45 | 0.04 | 0.005 | *-* | *AGPAT6* | up. (4) 5’ UTR (1) |
| 29 | 33 | MON | αs1-CN | 9224409 | 9748438 | 218 | 111 | 5 | **9571372** | 17.6 | 0.26 | 0.07 | 0.008 | *-* | *PICALM* | intron (2) |
| 29 | 33 | HOL | αs1-CN | 9061609 | 9658108 | 18 | 4 | 2 | 9563396 | 11.7 | 0.38 | 0.06 | 0.009 | *-* | *PICALM* | up. |
| 29 | 33 | multi | αs1-CN | 9542167 | 9577372 | 9 | 0 | 0 | **9571372** | 35.6 | 0.29 | 0.06 | 0.005 | *-* | *-* | - |
| 29 | 34 | multi | PC | 43444435 | 44954627 | 83 | 57 | 18 | 44180314 | 8.6 | 0.05 | 0.05 | 0.009 | *-* | *CDC42EP2* | intron (2) |

^a^Positions of the variants with the maximum –log_10_(*P*) or bounds of the interval, separated by a hyphen, that contained all the top variants if more than two variants were in the top

^b^xxxxx for ENSBTAG00000xxxxx

SE = standard error
