# Supplementary material for: Within-breed and multi-breed GWAS on imputed whole-genome sequence variants reveal candidate mutations affecting milk protein composition in dairy cattle
Source: Genet Sel Evol. 2017 Sep 18;49:68. doi: 10.1186/s12711-017-0344-z (PMC5604355; doi:10.1186/s12711-017-0344-z)
Supplement: Supplementary file 5 — Additional file 5: Table S5. Functional annotations of variants included within confidence intervals (± 100 kb) of the 34 QTL for each trait in the three within-breed Montbéliarde (MON), Normande (NOR), and Holstein (HOL) or in multi-breed analyses. [file 12711_2017_344_MOESM5_ESM.docx]

**Table S3.** Functional annotations of variants included within confidence intervals (±100kb) of the 34 QTL for each trait in the three within-breed Montbéliarde (MON), Normande (NOR), and Holstein (HOL) or in multi-breed analyses

| **Breed** | **Functional annotation** | **PC** | **α-LA** | **β-LG** | **αs1-CN** | **αs2-CN** | **β-CN** | **κ-CN** | **Σ-CN** | **Σ-WP** |
| --- | --- | --- | --- | --- | --- | --- | --- | --- | --- | --- |
| MON | 3’ UTR |  |  | 3 |  |  |  | 8 |  |  |
|  | 5’ UTR |  | 2 | 2 | 2 |  | 2 | 2 | 2 | 2 |
|  | downstream | 2 | 79 | 111 | 120 | 10 | 84 | 138 | 83 | 83 |
|  | intergenic | 4 | 217 | 161 | 677 | 7 | 169 | 163 | 58 | 58 |
|  | intronic | 129 | 114 | 102 | 390 | 47 | 85 | 96 | 53 | 53 |
|  | missense | 1 | 2 | 3 | 8 | 1 | 2 | 4 | 2 | 2 |
|  | splice region variant |  | 1 | 2 | 2 |  | 1 | 2 | 1 | 1 |
|  | synonymous | 1 | 3 | 4 | 8 | 1 | 3 | 4 | 3 | 3 |
|  | upstream | 19 | 56 | 66 | 96 | 19 | 60 | 73 | 60 | 60 |
|  | **Total** | **156** | **474** | **454** | **1303** | **85** | **406** | **490** | **262** | **262** |
| NOR | 3’ UTR | 2 |  |  |  | 1 |  | 11 |  |  |
|  | 5’ UTR | 5 | 1 | 2 | 3 | 1 | 6 | 5 | 2 | 2 |
|  | downstream | 79 | 23 | 103 | 154 | 51 | 140 | 232 | 103 | 103 |
|  | intergenic | 131 | 95 | 63 | 243 | 113 | 194 | 500 | 63 | 63 |
|  | intronic | 295 | 387 | 58 | 134 | 81 | 249 | 456 | 58 | 58 |
|  | missense | 15 | 9 | 5 | 10 | 1 | 14 | 18 | 5 | 5 |
|  | non coding exon |  |  |  |  | 2 |  |  |  |  |
|  | splice region variant | 5 | 4 | 1 | 5 |  | 6 | 5 | 1 | 1 |
|  | synonymous | 19 | 21 | 5 | 14 |  | 23 | 22 | 5 | 5 |
|  | upstream | 143 | 82 | 67 | 138 | 43 | 158 | 244 | 67 | 67 |
|  | **Total** | **694** | **622** | **304** | **701** | **293** | **790** | **1493** | **304** | **304** |
| HOL | 3’ UTR | 19 |  |  | 7 | 13 | 16 | 11 | 3 |  |
|  | 5’ UTR | 1 |  | 2 | 2 | 1 | 2 | 3 | 3 | 2 |
|  | downstream | 230 | 7 | 89 | 150 | 179 | 199 | 219 | 114 | 89 |
|  | inframe insertion |  |  |  |  |  |  |  | 1 |  |
|  | intergenic | 413 | 590 | 46 | 410 | 216 | 480 | 380 | 82 | 59 |
|  | intronic | 405 | 620 | 31 | 130 | 213 | 183 | 166 | 116 | 56 |
|  | missense | 17 |  | 2 | 4 | 12 | 6 | 15 | 14 | 2 |
|  | splice acceptor | 1 |  |  |  | 1 | 1 |  |  |  |
|  | splice region variant | 4 | 3 | 1 | 1 | 3 | 1 | 4 | 4 | 1 |
|  | synonymous | 27 | 5 | 4 | 5 | 24 | 5 | 29 | 10 | 4 |
|  | upstream | 82 | 11 | 56 | 65 | 56 | 83 | 106 | 88 | 65 |
|  | **Total** | **1199** | **1236** | **231** | **774** | **718** | **976** | **933** | **435** | **278** |
| Multi | 3’ UTR | 1 |  | 6 | 8 | 4 | 7 | 8 | 1 |  |
|  | 5’ UTR | 2 | 2 | 2 | 2 | 3 | 3 | 3 | 1 |  |
|  | downstream | 48 | 112 | 125 | 190 | 170 | 214 | 225 | 43 | 32 |
|  | intergenic | 82 | 89 | 172 | 802 | 121 | 315 | 343 | 41 | 6 |
|  | intronic | 163 | 461 | 109 | 402 | 136 | 170 | 197 | 83 | 8 |
|  | missense | 5 | 9 | 5 | 9 | 6 | 7 | 8 | 3 | 4 |
|  | non coding exon |  |  |  |  | 2 | 2 | 2 |  |  |
|  | splice region variant | 2 | 2 | 1 | 2 | 2 | 2 | 2 |  |  |
|  | synonymous | 10 | 13 | 5 | 18 | 12 | 9 | 15 | 3 | 1 |
|  | upstream | 58 | 75 | 67 | 93 | 114 | 120 | 113 | 41 | 4 |
|  | **Total** | **371** | **763** | **492** | **1526** | **570** | **849** | **916** | **216** | **55** |
