# Supplementary material for: Within-breed and multi-breed GWAS on imputed whole-genome sequence variants reveal candidate mutations affecting milk protein composition in dairy cattle
Source: Genet Sel Evol. 2017 Sep 18;49:68. doi: 10.1186/s12711-017-0344-z (PMC5604355; doi:10.1186/s12711-017-0344-z)
Supplement: Supplementary file 6 — Additional file 6: Figure S1. −log10(P) plotted against the position of variants detected by GWAS (in grey) and conditional GWAS (GWAS_COJO; in blue) a On BTA1, b BTA2, c BTA5, d BTA11, e BTA14, f BTA20, g BTA27 and h BTA29 [file 12711_2017_344_MOESM6_ESM.docx]

**Figure S1:** -log(*P*-value) plotted against the position of variants detected by GWAS (in grey) and conditional GWAS (GWAS_COJO; in blue)

**a) On BTA1, in Montbéliarde analysis for the αs1-CN phenotype, with the fixed effect of**

a1) the TOP1 variant detected in within-breed Montbéliarde GWAS, at 144,397,274 bp

a2) the TOP1 variant detected in multibreed GWAS, at 144,398,814 bp

a3) the most probable candidate variant detected by Kemper et al (2015), at 144,414,936 bp, ranked 101^st^ in multibreed GWAS


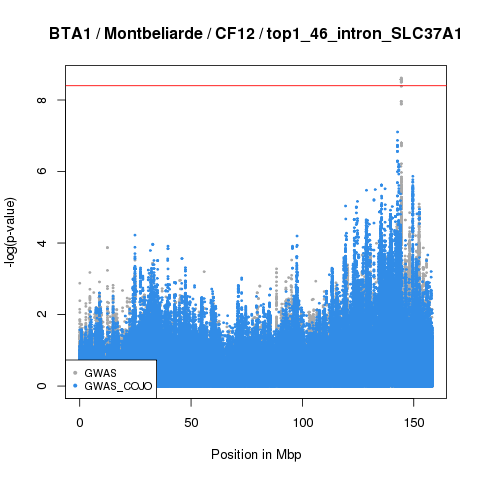

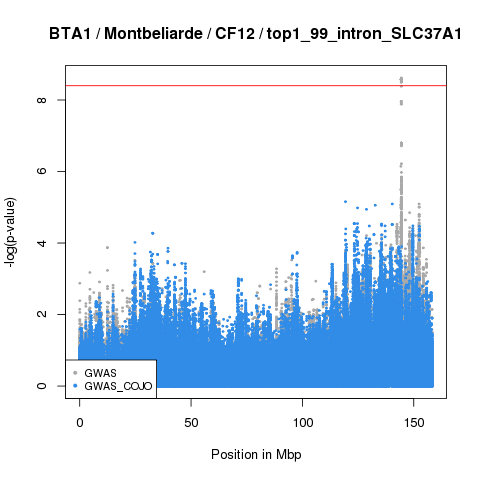

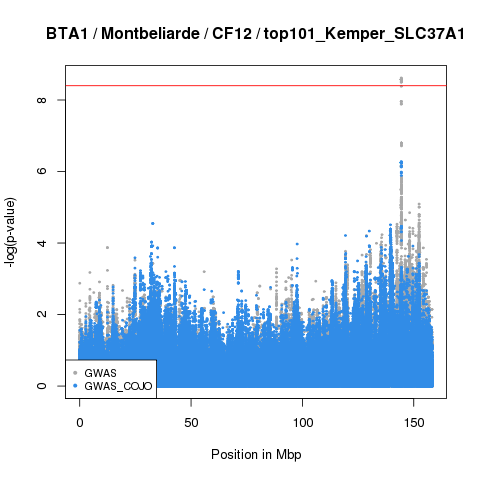


a1)

a3)

a2)

**b) On BTA2, in Montbéliarde, Normande, and Holstein analyses for the αs2-CN** **phenotype,** with the fixed effect of the TOP1 intronic variant detected in the within-breed Normande GWAS, at 131,806,882 bp

**
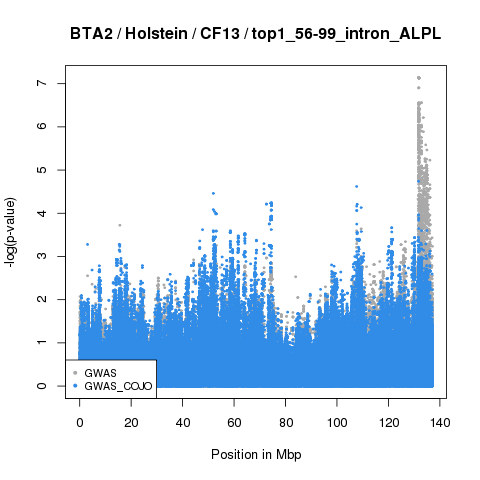

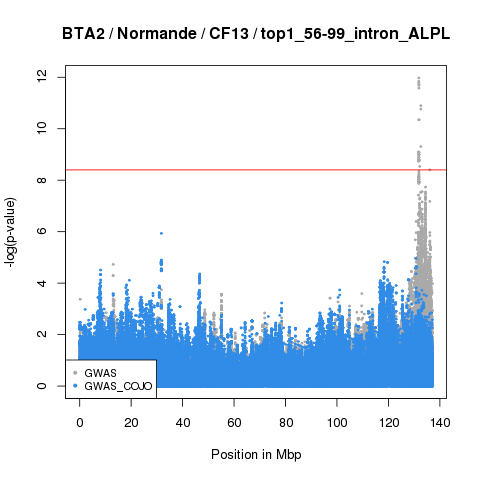

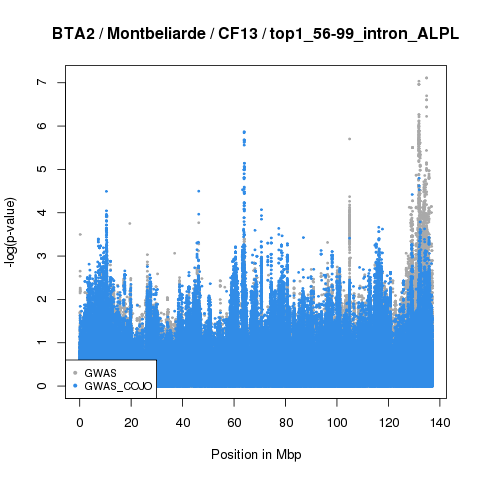
**

Montbéliarde

Holstein

Normande

**c) On BTA5, in Holstein analysis for the PC phenotype, with the fixed effect of**

c1) the TOP1 upstream variant detected in the multi-breed GWAS, at 93,950,211 bp

c2) the TOP2 5’-UTR or TOP3 upstream variants detected in the multi-breed GWAS, at 93,950,116 and 93,950,288 bp

c3) the upstream variant detected by Raven et al. (2015) at 93,951,731 bp and ranked 23^rd^ in the multi-breed GWAS

c4) the intronic variant detected by Iso-Touru et al. (2016) at 93,945,694 bp, outside of the confidence interval of the QTL detected in the multi-breed GWAS


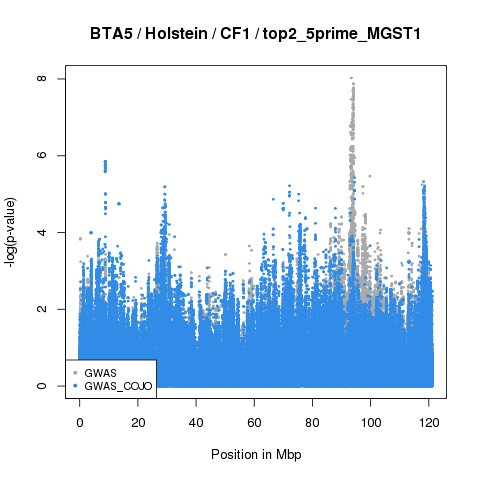

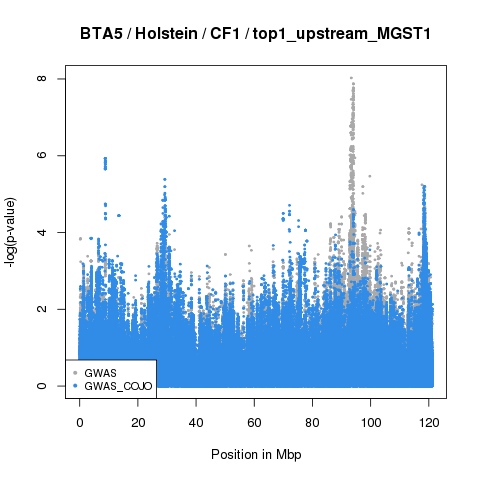

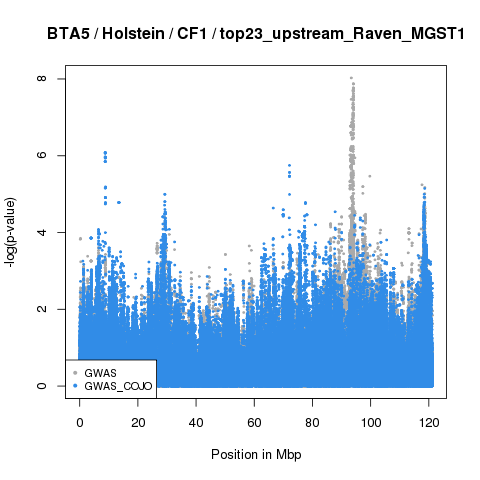

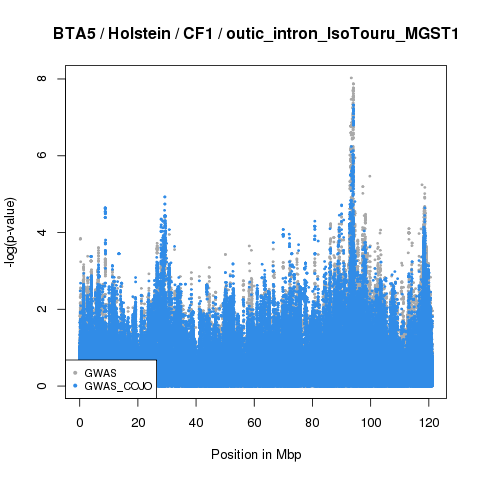


c2)

c1)

c4)

c3)

**d) On BTA11, in Montbéliarde, Normande, and Holstein analyses for the β-LG phenotype, with the fixed effect of**

d1) the missense PAEP mutations that cause the A and B protein variants, at 103,303,475 and 103,304,757 bp (Ganai et al., 2009)

d2) the upstream PAEP variant (103,298,431 bp) ranked in the TOP6 in within- and multi-breed analyses


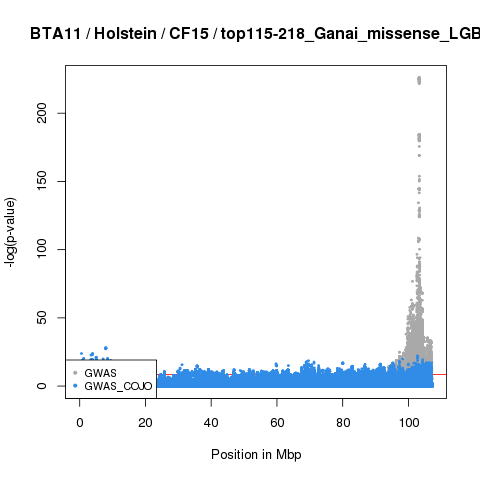

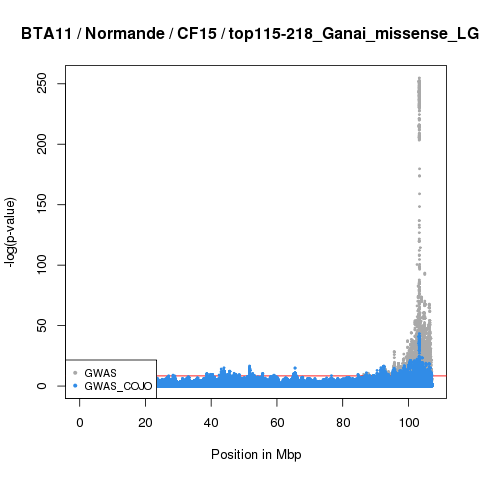

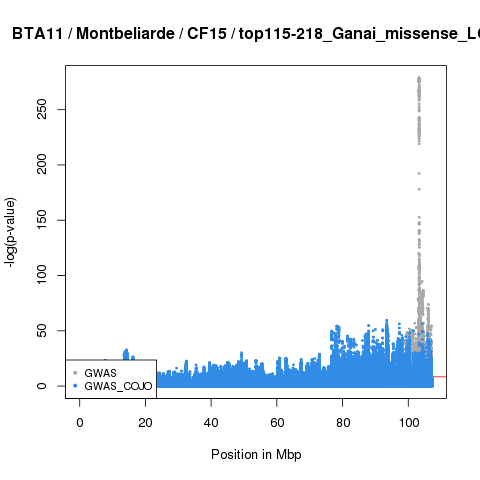
d1)

Holstein

Normande

Montbéliarde


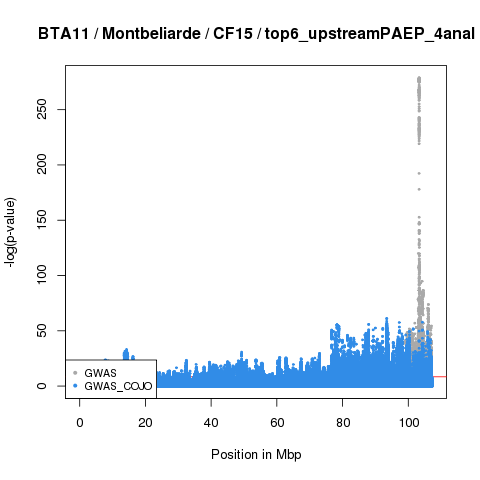

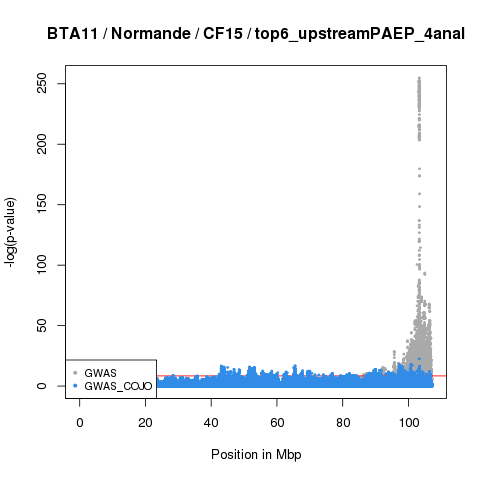

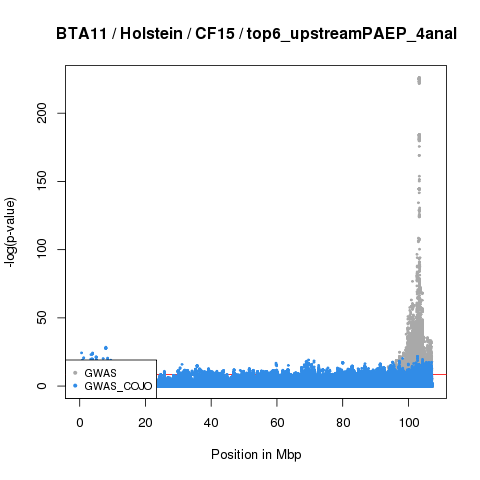
d2)

Montbéliarde

Holstein

Normande

**e) On BTA14, in Normande and Holstein analyses, with the fixed effect of**

e1) the causative K232A *DGAT1* variant for the κ-CN phenotype, at 1,802,266 bp (Grisart et al., 2001)

e2) the missense *BOP1* variant (1,842,678 bp), ranked 1^st^ in the NOR analysis for the κ-CN phenotype

e3) one of the three missense *RECQL4* variants in complete LD (1,617,841, 1,618,978, and 1,619,555 bp), ranked 3^rd^ in the HOL analysis for the κ-CN phenotype

e4) the missense *MROH1* variant (1,878,165 bp), ranked 14^th^ in the HOL analysis for the αs2-CN phenotype


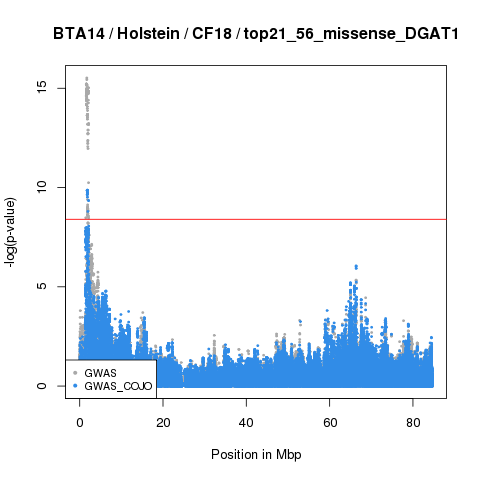

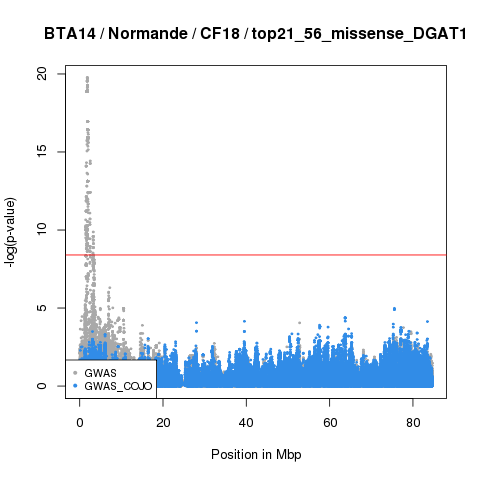
e1)

Holstein

Normande


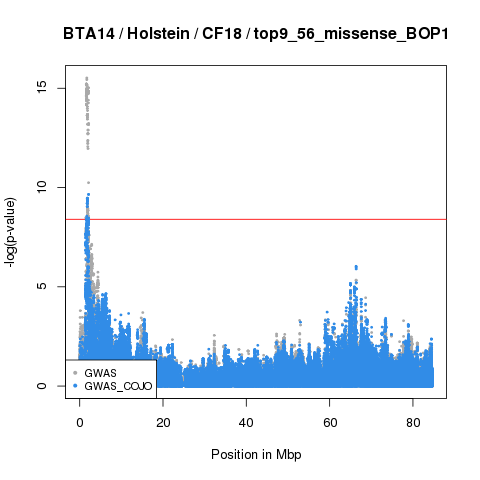

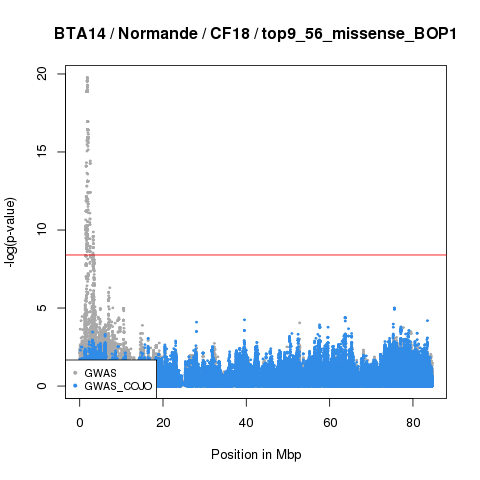
e2)

Normande

Holstein


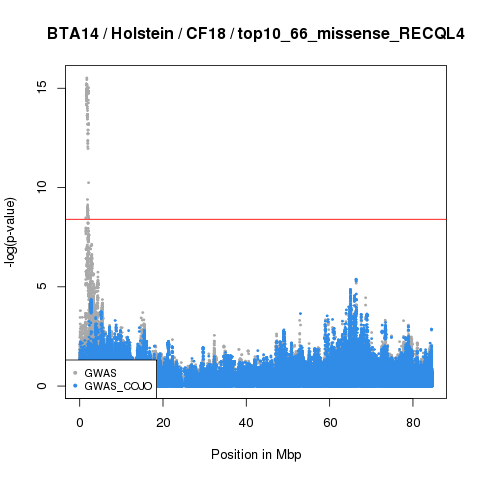

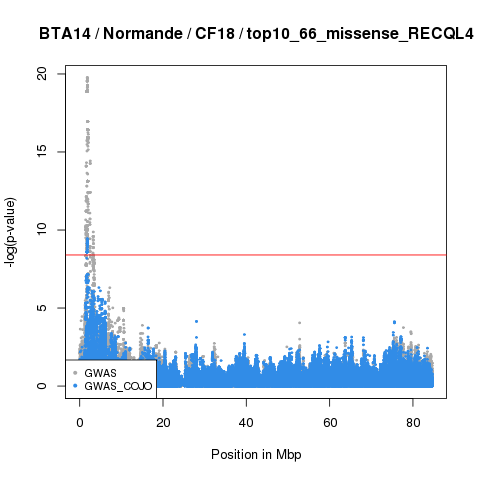
e3)

Normande

Holstein


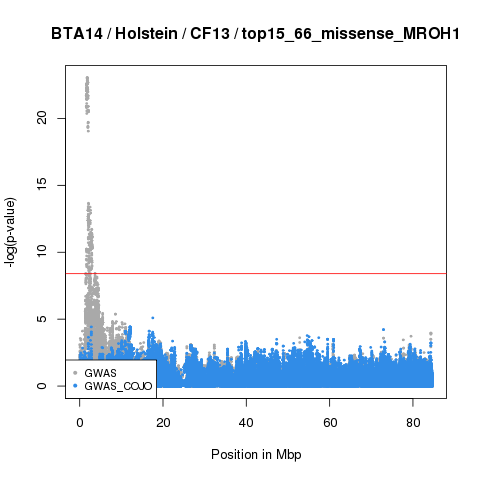

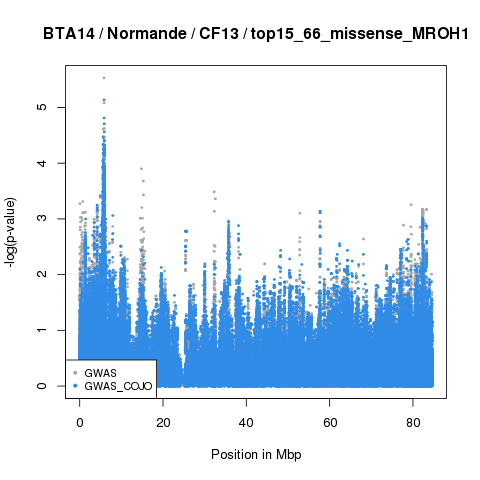


e4)

Normande

Holstein

**f) On BTA20, for the α-LA phenotype, with the fixed effect of the most significant variant, located in the *ANKH* gene in Montbéliarde (58,446,550 bp), Normande (58,422,697 bp), and Holstein (58,491,204 bp) analyses**

**
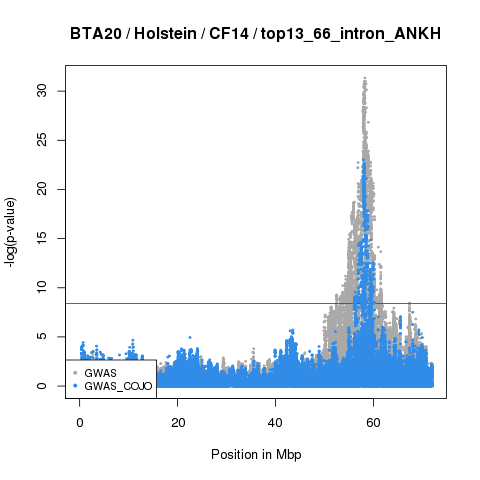

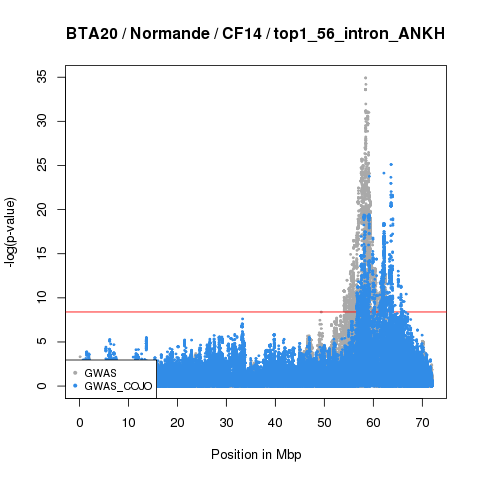

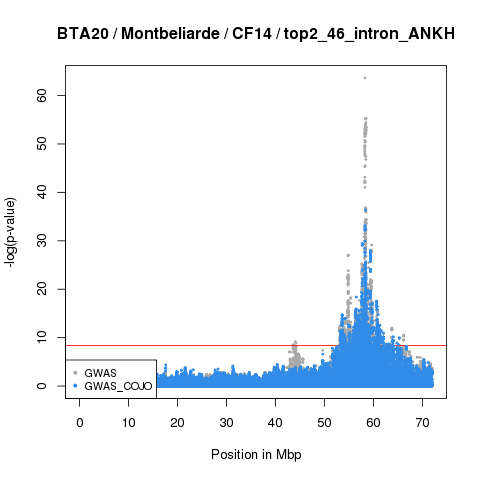
**

Holstein

Normande

Montbéliarde

**g) On BTA27, for the κ-CN phenotype, with the fixed effect of one of the five most significant variants located in the *AGPAT6* gene (36,209,319, 36,211,252, 36,211,258, 36,211,708, and 36,212,352 bp) in Montbéliarde, Normande, Holstein, and multi-breed analyses**

**
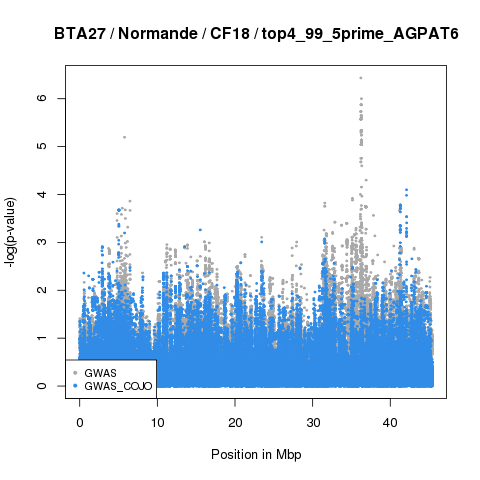

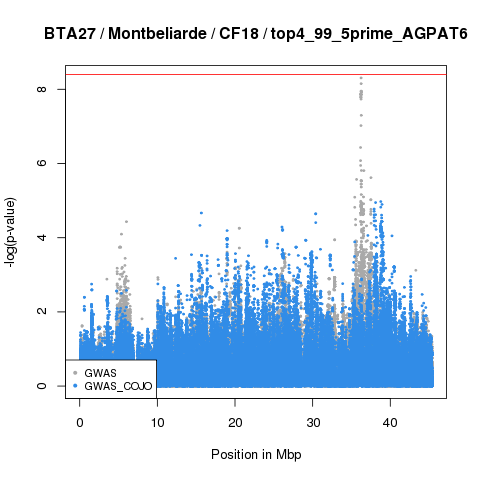
**

Normande

Montbéliarde

**
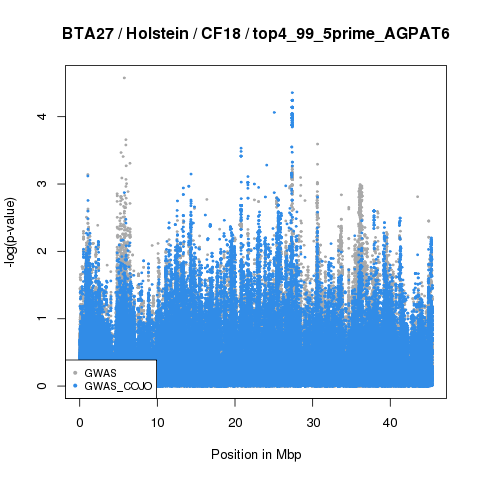

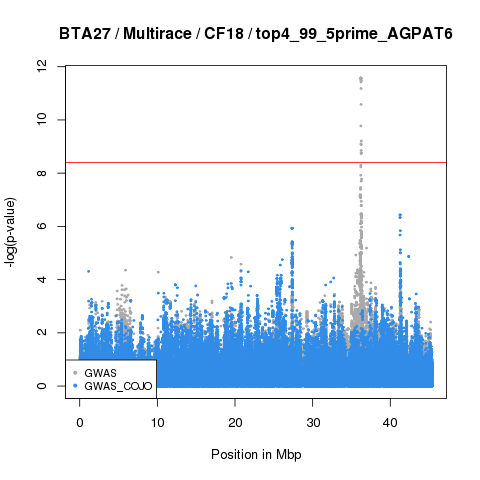
**

Holstein

Multi-breed

**h) On BTA29, in Montbéliarde, Normande, and Holstein analyses for the αs1-CN phenotype, with the fixed effect of**

h1) the intronic PICALM variants, at 9,651,065 and 9,656,439 bp, ranked 11^th^ in the MON analysis

h2) the upstream PICALM variant, at 9,611,304 bp, ranked 10^th^ in the HOL analysis

**
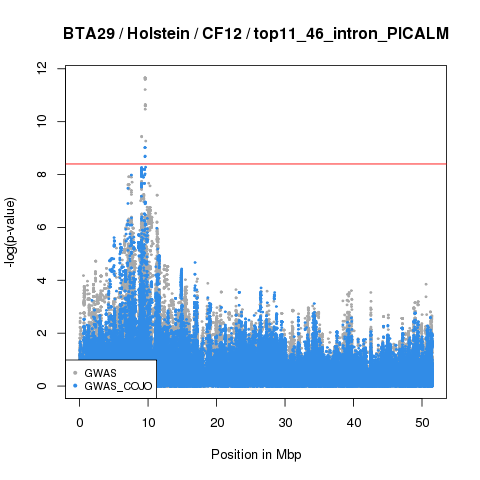

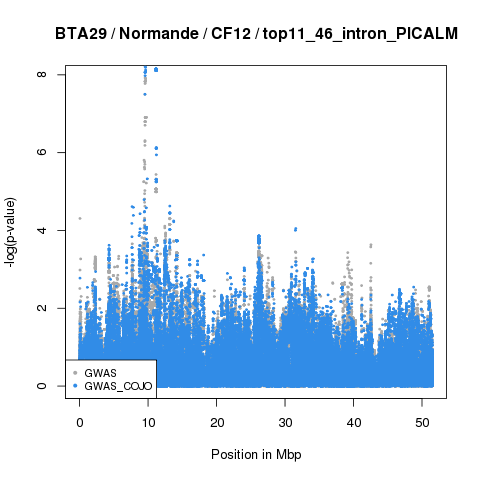

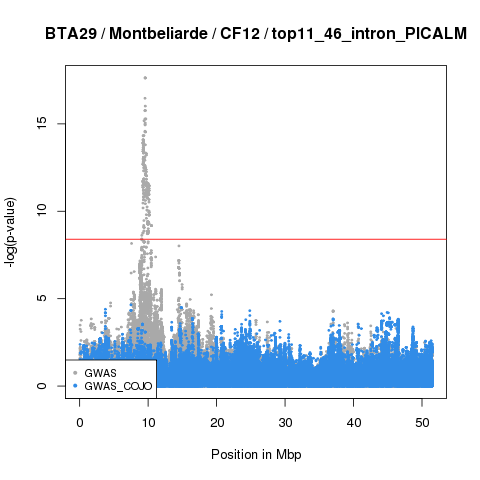
**

**
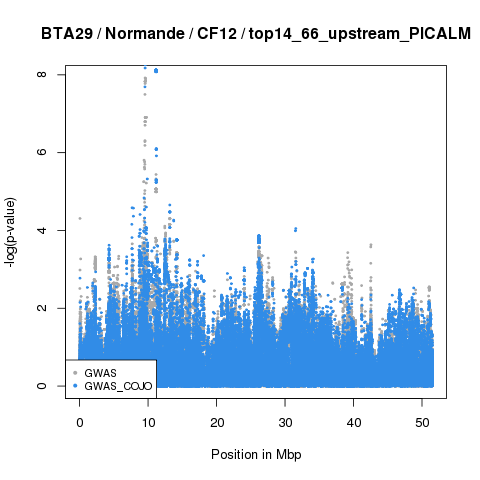

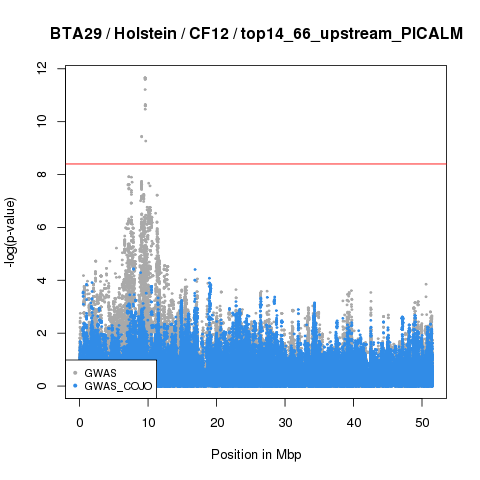

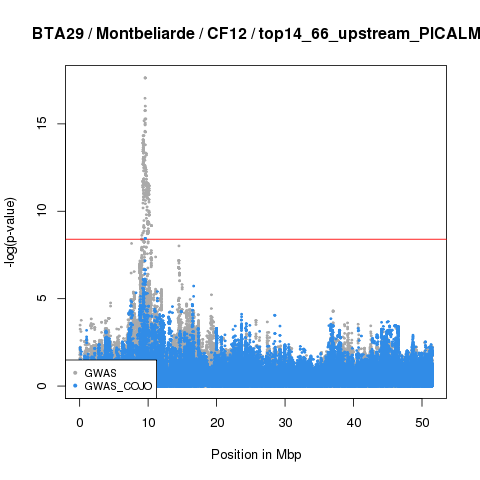
**
